# Supplementary material for: Comprehensive characterization of PTEN mutational profile in a series of 34,129 colorectal cancers
Source: Nat Commun. 2022 Mar 25;13:1618. doi: 10.1038/s41467-022-29227-2 (PMC8956741; doi:10.1038/s41467-022-29227-2)
Supplement: Supplementary file 1 — Supplementary Information [file 41467_2022_29227_MOESM1_ESM.pdf]

Supplementary Table 1. Age distribution of patients with CRC by site, sex, and MS status (relevant to Figure 1c, Supplementary Figure 1a,b)

	Age distribution of patients with CRC by site					
**site**	Q0	Q1	median	Q3	Q4	mean
**colon**	8	50	59	68	89	58.9
**rectum**	14	49	57	66	89	57.1
	Age distribution of patients with CRC by sex					
**sex**	Q0	Q1	med	Q3	Q4	mean
**F**	12	49	59	68	89	58.4
**M**	8	50	59	68	89	58.7
Age distribution of patients with CRC by MS cohort						
**MS cohort**	Q0	Q1	median	Q3	Q4	mean
**MT-H**	8	51	64	75	89	62.4
**MT-L**	8	50	59	67	89	58.4
**MSS htmb**	16	44	55	65	87	53.8

Supplementary Table 2. Sex distribution of patients, by MS status (relevant to Figure 1d)
Significance was calculated using 2-sample test for equality of proportions with continuity correction (prop.test function in R).

MS cohort	sex	count	fraction	comparison	p-value
**MT-H**	F	812	0.507	MT-H to MT-L	2.92E-06
**MT-H**	M	791	0.493		
**MT-L**	F	14389	0.447	MT-L to MSS htmb	0.000377
**MT-L**	M	17823	0.553		
**MSS htmb**	F	80	0.331	MSS htmb to MT-H	4.73E-07
**MSS htmb**	M	162	0.669		

Supplementary Table 3. Distribution of tumor subsites, by MS status (relevant to Figure 1e)
Significance was calculated using 2-sample test for equality of proportions with continuity correction (prop.test function in R).

MS cohort	site	count	fraction	comparison	p-value
**MT-H**	colon	1526	0.952	MT-H to MT-L	5.60E-37
**MT-H**	rectum	77	0.048		
**MT-L**	colon	26807	0.832	MT-L to MSS htmb	0.561
**MT-L**	rectum	5426	0.168		
**MSS htmb**	colon	206	0.848	MSS htmb to MT-H	7.90E-10
**MSS htmb**	rectum	37	0.152		

Supplementary Table 4. Prevalence of PTEN mutations in CRC cohorts (relevant to Figure 2b)

Column	Description
**MS cohort**	status assigned by integrating microsatellite instability and TMB.
**samples**	counts of samples
**single_pten**	counts of samples carrying a single PTEN alterations
**mult_pten**	counts of samples carrying multiple PTEN alterations
**any_pten**	counts of samples carrying at least one PTEN alteration
**fract_single**	fraction of samples carrying a single PTEN alterations
**fract_mult**	fraction of samples carrying multiple PTEN alterations
**fraction_any**	fraction of samples carrying at least one PTEN alteration

Confidence intervals for a population proportion, as well as significance of difference between proportions (using 2-sample test for equality of proportions with continuity correction) were calculated based on the data for any PTEN alterations, using prop.test function in R.

		Samples with PTEN alterations, count			Samples with PTEN alterations, fraction			Confidence intervals comb		comparisons	
MS cohort	patients	Single	Mult	Any	Single	Mult	Any	lower	upper	groups	p-value
**MT-H**	1603	211	189	400	0.132	0.118	0.250	0.229	0.272	MT-H to MT-L	8.80E-132
**MT-L**	32233	2301	137	2438	0.071	0.004	0.076	0.073	0.079	MT-L to MSS htmb	5.29E-104
**MSS htmb**	243	43	67	110	0.177	0.276	0.453	0.389	0.518	MSS htmb to MT-H	6.70E-11

Supplementary Table 5. Prevalence of PTEN mutations in CRC cohorts by sex (relevant to Fig.2d)

MS cohort	sex	PTEN alt	PTEN wt	Fraction pten	ConfL	ConfU	p-value by sex
MT-H	F	211	600	0.260	0.2306	0.2921	0.3399
MT-H	M	188	601	0.238	0.2093	0.2699	
MT-L	F	1290	13099	0.090	0.0851	0.0945	2.00E-17
MT-L	M	1148	16675	0.064	0.0609	0.0681	
MSS htmb	F	28	52	0.350	0.2490	0.4656	0.0309
MSS htmb	M	82	80	0.506	0.4269	0.5851	

Supplementary Table 6. Prevalence of PTEN mutations in CRC cohorts by tumor subsite (relevant to Fig.2e)

MS cohort	sex	PTEN alt	PTEN wt	Fraction pten	ConfL	ConfU	p-value by site
MT-H	colon	381	1142	0.250	0.229	0.273	0.85
MT-H	rectum	18	59	0.234	0.148	0.347	
MT-L	colon	2138	24669	0.080	0.077	0.083	6.11E-10
MT-L	rectum	300	5126	0.055	0.049	0.062	
MSS htmb	colon	96	110	0.466	0.397	0.537	0.42
MSS htmb	rectum	14	23	0.378	0.229	0.552	

Supplementary Table 7. PTEN alteration trends by age and MS cohort (relevant to Fig.2f)

Logistic regression coefficients and p-values were calculated using the glm function in R

MS cohort	PTEN alt	wt	reg_coef	p_value
**MT-H**	400	1203	0.00067	0.32366
**MT-L**	2438	29795	0.00062	1.6E-07
**MSS htmb**	110	133	-0.0072	0.00067

Supplementary Table 8. PTEN alteration trends by age, MS cohort, and tumor subsite (relevant to Supplementary Fig.2, bdf)
Logistic regression coefficients and p-values were calculated using the glm function in R

Assigned	**site**	**PTEN alt**	**wt**	**reg_coef**	**p_value**
**MT-H**	colon	381	1142	0.000933	0.190803
**MT-H**	rectum	18	59	-0.005512	0.076489
**MT-L**	colon	2138	24669	0.000571	0.000014
**MT-L**	rectum	300	5126	0.000663	0.008633
**MSS htmb**	colon	96	110	-0.006711	0.003187
**MSS htmb**	rectum	14	23	-0.011004	0.050463

Supplementary Table 9. PTEN alteration trends by age, MS cohort and sex (relevant to Supplementary Fig.2, ceg)
Logistic regression coefficients and p-values were calculated using the glm function in R

Assigned	**sex**	**PTEN alt**	**wt**	**reg_coef**	**p_value**
**MT-H**	F	211	600	-0.000464	0.657156
**MT-H**	M	188	601	0.001416	0.135962
**MT-L**	F	1290	13099	0.000756	0.000051
**MT-L**	M	1148	16675	0.000555	0.000187
**MSS htmb**	F	28	52	-0.005393	0.112681
**MSS htmb**	M	82	80	-0.007459	0.005152

Supplementary Table 10. Major PTEN alteration classes, by MS cohort (relevant to Fig.2g)

**MS cohort**	**alteration_Class**	**count**	**fraction in cohort**
**MT-H**	deletion	1	0.00165
**MT-H**	missense_indel	177	0.292
**MT-H**	truncation	429	0.707
**MT-L**	amplification	1	0.000387
**MT-L**	deletion	1055	0.408
**MT-L**	missense_indel	640	0.248
**MT-L**	rearrangement	38	0.0147
**MT-L**	truncation	851	0.329
**MSS htmb**	deletion	5	0.0234
**MSS htmb**	missense_indel	132	0.617
**MSS htmb**	truncation	77	0.36

Supplementary Table 11. Major PTEN alteration classes, in MT-L and MT-H cohorts, by sex (relevant to Supplementary Fig.2h)

MS cohort	sex	alteration_Class	n_samples
**MT-H**	F	deletion	1
**MT-H**	F	missense & inframe indels	99
**MT-H**	F	truncation	208
**MT-H**	M	missense & inframe indels	78
**MT-H**	M	truncation	219
**MT-L**	F	deletion	569
**MT-L**	F	other	20
**MT-L**	F	missense & inframe indels	326
**MT-L**	F	truncation	452
**MT-L**	M	deletion	486
**MT-L**	M	other	19
**MT-L**	M	missense & inframe indels	314
**MT-L**	M	truncation	399

Supplementary Table 12. Major PTEN alteration classes, in MT-L and MT-H cohorts, by tumor subsite (relevant to Supplementary Fig.2i)

MS cohort	site	alteration_Class	n_samples
**MT-H**	colon	deletion	1
**MT-H**	colon	missense & inframe indels	168
**MT-H**	colon	truncation	410
**MT-H**	rectum	missense & inframe indels	9
**MT-H**	rectum	truncation	17
**MT-L**	colon	deletion	927
**MT-L**	colon	other	34
**MT-L**	colon	missense & inframe indels	553
**MT-L**	colon	truncation	756
**MT-L**	rectum	deletion	128
**MT-L**	rectum	other	5
**MT-L**	rectum	missense & inframe indels	87
**MT-L**	rectum	truncation	95

Supplementary Table 13. Major PTEN alteration classes, in MT-L and MT-H cohorts, by age (relevant to Supplementary Fig.2j)
In each of the MTL and MTH subsets, the samples with PTEN alterations were ranked by age and split into three equal groups.

Age group	alteration_Class	n_samples MTL	n_samples MTH
1	deletion	293	0
1	other	15	0
1	missense & inframe indels	164	45
1	truncation	256	146
2	deletion	347	0
2	other	10	0
2	missense & inframe indels	231	67
2	truncation	298	138
3	deletion	401	1
3	other	13	0
3	missense & inframe indels	237	68
3	truncation	287	137

Supplementary Table 14. PTEN post-translational modifications (relevant to Fig.3a)
References are given as PMIDs

Amino Acid	Modification	Ref.1	Ref.2	Ref.3	Ref.4	UNIPROT
K13	ubiquitination	17218261				YES
K63	ubiquitination	34416231				NO
C83	S-nitrosylation	21646525				NO
K125	acetylation	16829519				NO
K128	acetylation	16829519				NO
K163	acetylation	26279303				NO
S229	phosphorylation	15793569				NO
Y240	phosphorylation	30827889				NO
K254	SUMOylation	23888040	22713753			NO
K266	SUMOylation	23888040	22713753	23013792	22713753	NO
K289	ubiquitination	17218261				YES
T321	phosphorylation	15793569				NO
Y336	phosphorylation	19345329				YES
T366	phosphorylation	12297295	20940307			YES
S370	phosphorylation	12297295	20940307	11035045		YES
S380	phosphorylation	24706748				YES
T382	phosphorylation	11035045	24706748			YES
T383	phosphorylation	11035045	24706748			YES
S385	phosphorylation	11035045	12297295			YES
T398	phosphorylation	23888040				NO
T401	phosphorylation	10646847				YES
K402	acetylation	18757404				NO

Supplementary Table 15. PTEN single-residue hotspots, by MS/TMB cohort

Column	Description
**Counts_**	number of mutations in a given codon.
**as % in**	share of mutations in a given codon among all mutations in cohort
**found in cancerhotspots**	whether or not this hotspot was identified in the pancancer cohort, as available on cancerhotspots.org
**Cutoff** (top line):	cutoffs calculated using binomial distribution for the p-value 0.005

cutoff	**9**	**8**	**5**	**4**					
Codon	Counts **all**	Counts **MTL**	Counts **MTH**	Counts **MSS htmb**	as % **overall**	as % in **MTL**	as % in **MTH**	as % in **MSS htmb**	found in cancerhotspots
**130**	177	108	27	41	8%	8%	5%	20%	**Y**
**267**	133	3	129	0	6%	0%	22%	0%	**Y**
**173**	111	67	27	15	5%	5%	5%	7%	**Y**
**323**	96	20	76	0	5%	2%	13%	0%	**n**
**233**	95	65	27	2	4%	5%	5%	1%	**Y**
**319**	62	54	7	1	3%	4%	1%	0%	**n**
**299**	33	6	1	25	2%	0%	0%	12%	**n**
**157**	30	9	21	0	1%	1%	4%	0%	**n**
**7**	29	5	0	23	1%	0%	0%	11%	**n**
**268**	28	7	20	1	1%	1%	3%	0%	**Y**
**136**	24	21	3	0	1%	2%	1%	0%	**Y**
**33**	22	15	7	0	1%	1%	1%	0%	**n**
**68**	22	18	4	0	1%	1%	1%	0%	**Y**
**335**	22	16	6	0	1%	1%	1%	0%	**Y**
**27**	18	16	2	0	1%	1%	0%	0%	**Y**
**57**	18	9	9	0	1%	1%	2%	0%	**n**
**63**	18	3	13	1	1%	0%	2%	0%	**n**
**290**	18	5	12	1	1%	0%	2%	0%	**n**
**36**	17	14	2	1	1%	1%	0%	0%	**n**
**16**	16	15	1	0	1%	1%	0%	0%	**Y**
**165**	16	14	2	0	1%	1%	0%	0%	**Y**
**126**	15	13	2	0	1%	1%	0%	0%	**Y**
**93**	14	12	2	0	1%	1%	0%	0%	Y
**246**	14	12	1	1	1%	1%	0%	0%	n
**15**	12	7	1	4	1%	1%	0%	2%	n
**17**	12	12	0	0	1%	1%	0%	0%	n
**127**	12	11	1	0	1%	1%	0%	0%	Y
**134**	12	6	3	3	1%	0%	1%	1%	n
**155**	12	10	2	0	1%	1%	0%	0%	Y
**171**	12	12	0	0	1%	1%	0%	0%	Y
**274**	12	7	5	0	1%	1%	1%	0%	Y
**24**	11	8	2	1	1%	1%	0%	0%	Y
**111**	11	9	2	0	1%	1%	0%	0%	Y
**129**	11	8	3	0	1%	1%	1%	0%	Y
**142**	11	5	0	5	1%	0%	0%	2%	n
**320**	11	11	0	0	1%	1%	0%	0%	Y
**336**	11	8	0	3	1%	1%	0%	1%	Y
**66**	10	5	0	4	0%	0%	0%	2%	n
**92**	10	6	0	3	0%	0%	0%	1%	Y
**96**	10	3	7	0	0%	0%	1%	0%	n
**101**	10	9	1	0	0%	1%	0%	0%	**Y**
**105**	10	7	3	0	0%	1%	1%	0%	**Y**
**146**	10	6	4	0	0%	0%	1%	0%	**Y**
**164**	10	0	9	1	0%	0%	2%	0%	**n**
**211**	10	8	2	0	0%	1%	0%	0%	**n**
**277**	10	9	1	0	0%	1%	0%	0%	**Y**
**31**	9	6	2	0	0%	0%	0%	0%	**n**
**35**	9	9	0	0	0%	1%	0%	0%	**Y**
**38**	9	9	0	0	0%	1%	0%	0%	**Y**
**76**	9	5	4	0	0%	0%	1%	0%	**n**
**90**	9	1	8	0	0%	0%	1%	0%	**n**
**123**	9	8	0	1	0%	1%	0%	0%	**n**
**159**	9	8	1	0	0%	1%	0%	0%	**n**
**170**	9	6	2	0	0%	0%	0%	0%	**Y**
**124**	8	8	0	0	0%	1%	0%	0%	**Y**
**219**	8	8	0	0	0%	1%	0%	0%	**n**
**298**	8	8	0	0	0%	1%	0%	0%	**n**
**41**	7	3	0	4	0%	0%	0%	2%	**n**
**183**	6	2	0	4	0%	0%	0%	2%	**n**
**341**	8	3	0	4	0%	0%	0%	2%	**n**

Supplementary Table 16. Percent of major PTEN mutational single-residue hotspots, by MS/TMB cohort (relevant to Fig.3b)

	Hotspots MTL	Hotspots MTH	Hotspots MSS htmb
non-hotspot mut	49.1	29.4	36.5
R130	8.2	4.6	20.2
R173	5.1	4.6	7.4
R233	4.9	4.6	
T319	4.1		
K267		22.2	
N323		13.1	
E157		3.6	
D268		3.4	
E299			12.3
E7			11.3
other hotspots combined	32.7	14.5	12.3

Supplementary Table 17. Age trends for mutational signatures in MT-L and MT-H cohorts (relevant to Fig.4b). Regression coefficients and p-values were calculated using generalized linear model (glm function in R).

**MT-L**				
**signature**	PTEN alt	wt	**reg_coef**	**p_value**
**IDT_count**	60	32173	3.46E-05	0.07011
**PTEN_count**	2589	29791	0.000634	7.70E-07
**PTEN_nonsig**	2265	30074	0.000575	1.44E-06
**SBS1_count**	264	31972	2.42E-05	0.54931

**MT-H**				
**signature**	PTEN alt	wt	**reg_coef**	**p_value**
**IDT_count**	286	1370	-8.96E-05	0.90317
**PTEN_count**	608	1198	0.000719	0.52818
**PTEN_nonsig**	224	1403	0.000533	0.40021
**SBS1_count**	98	1506	0.000276	0.48141

Supplementary Table 18. Mutational signatures primarily associated with single-residue hotspots, by MS/TMB cohort (relevant to Fig.4c)

PTEN MT-L				PTEN MT-H				PTEN MSS htmb			
Codon	mut count	% of mut	Mut signature	Codon	mut count	% of mut	Mut signature	Codon	mut count	% of mut	Mut signature
130	108	8.2%	**SBS1**	267	129	22.2%	**IDT**	130	41	20%	**SBS10b/SBS1**
173	67	5.1%	**SBS1**	323	76	13.1%	**IDT**	299	25	12%	**SBS10a**
233	65	4.9%	**SBS1**	130	27	4.6%	**SBS1**	7	23	11%	**SBS10a**
319	54	4.1%	**repeat deletion**	173	27	4.6%	**SBS1**	173	15	7%	**SBS1**
136	21	1.6%		233	27	4.6%	**SBS1**	142	5	2%	**SBS10b/SBS1**
323	20	1.5%	**IDT**	157	21	3.6%	**IDT**	15	4	2%	**SBS10b**
68	18	1.4%		268	20	3.4%	**IDT**	41	4	2%	**SBS10a**
27	16	1.2%		63	13	2.2%	**IDT**	66	4	2%	
335	16	1.2%	**SBS1**	290	12	2.1%	**IDT**	183	4	2%	**SBS10a**
16	15	1.1%		57	9	1.5%	**IDT**	341	4	2%	**SBS28**
33	15	1.1%	**repeat deletion**	164	9	1.5%	**IDT**				
36	14	1.1%		90	8	1.4%	**IDT**				
165	14	1.1%		33	7	1.2%	**repeat deletion**				
126	13	1.0%		96	7	1.2%					
17	12	0.9%		319	7	1.2%	**repeat deletion**				
93	12	0.9%		335	6	1.0%	**SBS1**				
171	12	0.9%		274	5	0.9%	**IDT**				
246	12	0.9%									
127	11	0.8%									
320	11	0.8%									
155	10	0.8%									
35	9	0.7%									
38	9	0.7%									
57	9	0.7%	**IDT**								
101	9	0.7%									
111	9	0.7%									
157	9	0.7%									
277	9	0.7%									
24	8	0.6%									
123	8	0.6%									
124	8	0.6%									
129	8	0.6%									
159	8	0.6%									
211	8	0.6%									
219	8	0.6%									

Supplementary Table 19. Truncations prior to aa 352, by MS/TMB cohort

MS cohort	Truncations 1:352	non-truncations	total	fraction of truncations	fraction of non-truncations
**MT-H**	669	1920	2589	0.26	0.74
**MT-L**	403	207	610	0.66	0.34
**MSS htmb**	71	143	214	0.33	0.67

Supplementary Table 20. PTEN single-residue mutation hotspots, only considering missense/indels, by MS/TMB cohort (relevant to Fig.5, Supplementary Figs 7ab, 9acde)

Codon	All cohorts, M/I only	MTL, M/I only	MT-H, M/I only	MSS htmb, M/I only
173	110	66	27	15
130	91	41	10	39
33	22	15	7	NA
136	20	17	NA	NA
68	16	12	4	NA
126	15	13	NA	NA
27	14	12	NA	NA
36	14	11	NA	NA
93	14	12	NA	NA
246	14	12	NA	NA
155	12	10	NA	NA
15	11	6	NA	4
24	11	8	NA	NA
142	11	NA	NA	5
165	11	10	NA	NA
127	10	9	NA	NA
129	10	8	NA	NA
134	10	NA	NA	NA
31	9	6	NA	NA
35	9	9	NA	NA
66	9	NA	NA	4
92	9	NA	NA	NA
96	9	NA	7	NA
123	9	8	NA	NA
159	9	8	NA	NA
38	8	8	NA	NA
47	8	6	NA	NA
101	8	7	NA	NA
124	8	8	NA	NA
170	8	NA	NA	NA
252	8	6	NA	NA
76	7	NA	4	NA
95	7	6	NA	NA
128	7	6	NA	NA
131	7	7	NA	NA
132	7	6	NA	NA
277	7	6	NA	NA
45	6	NA	4	NA
105	6	6	NA	NA
138	6	NA	NA	NA
171	6	6	NA	NA
234	NA	NA	4	NA
41	NA	NA	NA	4
183	NA	NA	NA	4
341	NA	NA	NA	4

Supplementary Table 21. Distribution of LPA scores by cohort (relevant to Fig.6a, Supplementary Fig. 10ab).

MS cohort	sex		site		total in cohort	box plot stats					comparison		
	**F**	**M**	**colon**	**rectum**		**low whisker**	**low box**	**median**	**high box**	**high whisker**	**Comp. groups**	**t.test**	**ks.test**
**MT-H**	131	101	220	12	232	-4.79	-3.49	-2.04	-1.26	0.41	MT-H to MT-L	4.75E-04	0.001
**MT-L**	481	447	812	116	928	-5.41	-3.58	-2.69	-1.43	1.73	MT-L to MSS htmb	5.57E-04	3.08E-10
**MSS htmb**	50	145	167	28	195	-5.69	-3.38	-2.04	-1.26	0.56	MSS htmb to MT-H	0.647	0.005

Supplementary Table 22. Distribution of abundance scores by cohort (relevant to Fig.6b, Supplementary Fig. 10cd).

MS cohort	sex		site		total in cohort	box plot stats					comparison		
	**F**	**M**	**colon**	**rectum**		**low whisker**	**low box**	**median**	**high box**	**high whisker**	**Comp. groups**	**t.test**	**ks.test**
**MT-H**	51	41	85	7	92	-0.08	0.29	0.33	0.70	1.24	MT-H to MT-L	0.8	0.3
**MT-L**	243	198	387	54	441	-0.12	0.25	0.33	0.80	1.31	MT-L to MSS htmb	0.5	2.84E-04
**MSS htmb**	16	68	73	11	84	-0.05	0.16	0.32	0.73	1.24	MSS htmb to MT-H	0.4	5.57E-05

Supplementary Table 23. Distribution of LPA and abundance scores based on hotspot status (relevant to Fig.6gh).

**boxplot stats**	**LPA non-hotspot**	**LPA hotspot**
sample size	346	582
low whisker	-5.685	-5.41
low box	-3.488	-3.579
median	-2.2255	-2.472
high box	-0.718	-1.484
high whisker	1.727	1.295
	**Comparison (p-value)**	
ks.test	6.84E-07	
t.test	0.003083	
**boxplot stats**	**abundance non-hotspot**	**abundance hotspot**
sample size	181	260
low whisker	-0.124	-0.081
low box	0.21	0.255
median	0.481	0.29
high box	0.8355	0.64
high whisker	1.312	1.205
	**Comparison (p-value)**	
ks.test	0.0001618	
t.test	0.04735	

Supplementary Table 21. Distribution of LPA scores by cohort (relevant to Fig.6a, Supplementary Fig. 10ab). Statistical significance was calculated using Welch's unequal variances t-test and Kolmogorov-Smirnov test.

**MS cohort**	**sex**		**site**		total in cohort	**box plot stats**					**comparison**		
	**F**	**M**	**colon**	**rectum**		**low whisker**	**low box**	**median**	**high box**	**high whisker**	**Comp. groups**	**t.test**	**ks.test**
**MT-H**	127	96	211	12	223	-4.8	-3.5	-2	-1.3	0.4	MT-H to MT-L	4.75E-04	0.001
**MT-L**	475	440	800	115	915	-5.4	-3.6	-2.7	-1.4	1.7	MT-L to MSS htmb	5.57E-04	3.08E-10
**MSS htmb**	50	145	167	28	195	-5.7	-3.4	-2	-1.3	0.5	MSS htmb to MT-H	0.647	0.005

Supplementary Table 22. Distribution of abundance scores by cohort (relevant to Fig.6b, Supplementary Fig. 10cd). Statistical significance was calculated using Welch's unequal variances t-test and Kolmogorov-Smirnov test.

**MS cohort**	**sex**		**site**		total in cohort	**box plot stats**					**comparison**		
	**F**	**M**	**colon**	**rectum**		**low whisker**	**low box**	**median**	**high box**	**high whisker**	**Comp. groups**	**t.test**	**ks.test**
**MT-H**	88	64	141	11	152	-0.1	0.3	0.3	0.7	1.2	MT-H to MT-L	0.8	0.3
**MT-L**	325	300	549	76	625	-0.1	0.2	0.3	0.8	1.3	MT-L to MSS htmb	0.5	2.84E-04
**MSS htmb**	30	106	117	19	136	-0.1	0.2	0.3	0.7	1.2	MSS htmb to MT-H	0.4	5.57E-05

Supplementary Table 23. Distribution of LPA and abundance scores based on hotspot status (relevant to Fig.6gh). Statistical significance was calculated using Welch's unequal variances t-test and Kolmogorov-Smirnov test.

**boxplot stats**	**LPA non-hotspot**	**LPA hotspot**	**abundance non-hotspot**	**abundance hotspot**
sample size	346	582	181	260
low whisker	-5.685	-5.41	-0.124	-0.081
low box	-3.488	-3.579	0.21	0.255
median	-2.2255	-2.472	0.481	0.29
high box	-0.718	-1.484	0.8355	0.64
high whisker	1.727	1.295	1.312	1.205
Comparison (p-value)				
ks.test	6.84E-07		0.0001618	
t.test	0.003083		0.04735	

Supplementary Table 24. Comparison of copy number alteration profiles, by MS cohorts, subsite, and sex (p-values); relevant to Fig.7a and Supplementary Fig. 12. Statistical significance was calculated using two-sided Fisher exact test.

MS cohorts

Group	p_value
MT-L vs MT-H	0.0002
MSS_htmb vs MT-H	0.0792
MSS_htmb vs MT-L	0.0002

Sex (M vs F)

Group	p_value
MT-L	0.7942
MT-H	0.0148
MSS_htmb	0.3919

**Site (colon vs rectum)**	
Group	p_value
MT-L	0.1338
MT-H	0.1644
MSS_htmb	0.0002

Supplementary Table 25. Co-occurrence between PTEN mutations, by cohort Expected co-occurrence and the p-values for the actual co-occurrence were calculated using binominal distribution model

	**MT-L**				**MT-H**				**MT-L htmb**			
	all point mutations		LOF mutations only		all point mutations		LOF mutations only		all point mutations		LOF mutations only	
mut #	log2 (ratio)	p-value	log2 (ratio)	p-value	log2 (ratio)	p-value	log2 (ratio)	p-value	log2 (ratio)	p-value	log2 (ratio)	p-value
0	0.00328	0.955	0.00335	0.968	0.131	0.213	0.141	0.182	0.422	0.072	0.476	0.0452
1	-0.143	0.063	-0.172	0.038	-0.956	5.98E-10	-1.16	2.87E-14	-1.23	6.77E-05	-1.86	7.07E-07
2 or more	1.56	2.60E-08	1.9	3.63E-10	1.07	7.86E-08	1.22	2.40E-09	0.366	0.253	0.521	0.107

Supplementary Table 26. TMB distribution, by MS cohorts and PTEN mutation multiplicity. Related to Supplementary Figure 13.

box plot stats	MT-H mult	MT-H single	MT-L mult	MT-L single	MSS htmb mult	MSS htmb single
low whisker	11.3	15	0	0	17.5	16.3
low box	34.8	33.8	2.6	2.5	117.9	45.1
median	49.6	46.3	4.8	3.8	195	148.8
high box	63.8	63.9	7	6.1	275.7	256
high whisker	106.3	105.3	13.2	11.3	474.1	553.3

Supplementary Table 27. Co-occurrence or mutual exclusion between alterations in PTEN and genes shown, in the complete cohort (for any alteration). Statistical significance was calculated using two-sided Fisher exact test.

Second gene (gene B)	APC	KRAS	SMAD4	TP53	PIK3CA
Neither	6551	15464	24809	6844	25267
PTEN_not_ B	668	1286	2208	1102	2197
B_not_ PTEN	24612	15699	6354	24319	5896
Both	2298	1680	758	1864	769
log2 (odds ratio)	-0.1271	0.3638	0.42264	-1.0709	0.58495
p-value	0.05671	6.97E-11	9.96E-11	5.96E-71	5.73E-19
95% conf, upper limit	0.00546	0.47459	0.54895	-0.9552	0.71118
95% conf, lower limit	-0.2583	0.25328	0.29522	-1.186	0.45769

Supplementary Table 28. Co-occurrence or mutual exclusion between alterations in PTEN and genes shown (relevant to Fig.8ad). Statistical significance was calculated using two-sided Fisher exact test.

Gene_A	Gene_B	MS cohort	log2_odds	p_val	95% conf., upper	95% conf., bottom
PTEN_lof	APC	MT-L	-0.08	0.64728867	0.26	-0.40
PTEN_lof	KRAS	MT-L	0.86	2.30E-10	1.14	0.59
PTEN_lof	SMAD4	MT-L	0.45	0.00297895	0.75	0.15
PTEN_lof	TP53	MT-L	-1.38	8.82E-23	-1.11	-1.65
PTEN_lof	APC	MT-H	-0.20	0.45950189	0.35	-0.75
PTEN_lof	KRAS	MT-H	-0.38	0.23290731	0.23	-1.01
PTEN_lof	SMAD4	MT-H	0.20	0.59479284	0.94	-0.60
PTEN_lof	TP53	MT-H	-0.11	0.77358084	0.46	-0.69
PTEN_lof	APC	MSS htmb	4.07	7.11E-05	9.44	1.43
PTEN_lof	KRAS	MSS htmb	0.37	0.3956815	1.24	-0.50
PTEN_lof	SMAD4	MSS htmb	0.84	0.05278208	1.73	-0.04
PTEN_lof	TP53	MSS htmb	-0.01	1	0.89	-0.89
PTEN_deletion	APC	MT-L	-0.16	0.14652764	0.06	-0.38
PTEN_deletion	KRAS	MT-L	0.29	0.00172171	0.47	0.11
PTEN_deletion	SMAD4	MT-L	0.56	6.98E-08	0.76	0.36
PTEN_deletion	TP53	MT-L	-0.34	0.00112935	-0.13	-0.55
PTEN_presence	PIK3CA	all comb.	0.58	5.73E-19	0.71	0.46
PTEN_presence	PIK3CA	MT-L	0.24	1.50E-03	0.39	0.09
PTEN_point	PIK3CA	MT-L	0.68	1.11E-06	0.94	0.40
PTEN_lof	PIK3CA	MT-L	0.69	1.06E-05	0.98	0.38
PTEN_deletion	PIK3CA	MT-L	-0.49	1.08E-04	-0.24	-0.76
PTEN_lof	PIK3CA	MT-H	-0.08	7.79E-01	0.47	-0.65
PTEN_lof	PIK3CA	MSS htmb	2.03	3.00E-05	3.21	0.97

Supplementary references

1. Chang MT*, et al.* Accelerating Discovery of Functional Mutant Alleles in Cancer. *Cancer Discov* **8**, 174-183 (2018).

2. Chao JT*, et al.* A Premalignant Cell-Based Model for Functionalization and Classification of *PTEN* Variants. *Cancer Res* **80**, 2775-2789 (2020).

3. Hu Q*, et al.* LncRNAs-directed *PTEN* enzymatic switch governs epithelial-mesenchymal transition. *Cell Res* **29**, 286-304 (2019).

4. Gong L*, et al.* Nuclear *PTEN* tumor-suppressor functions through maintaining heterochromatin structure. *Cell Cycle* **14**, 2323-2332 (2015).

5. Post KL*, et al.* Multi-model functionalization of disease-associated *PTEN* missense mutations identifies multiple molecular mechanisms underlying protein dysfunction. *Nat Commun* **11**, 2073 (2020).

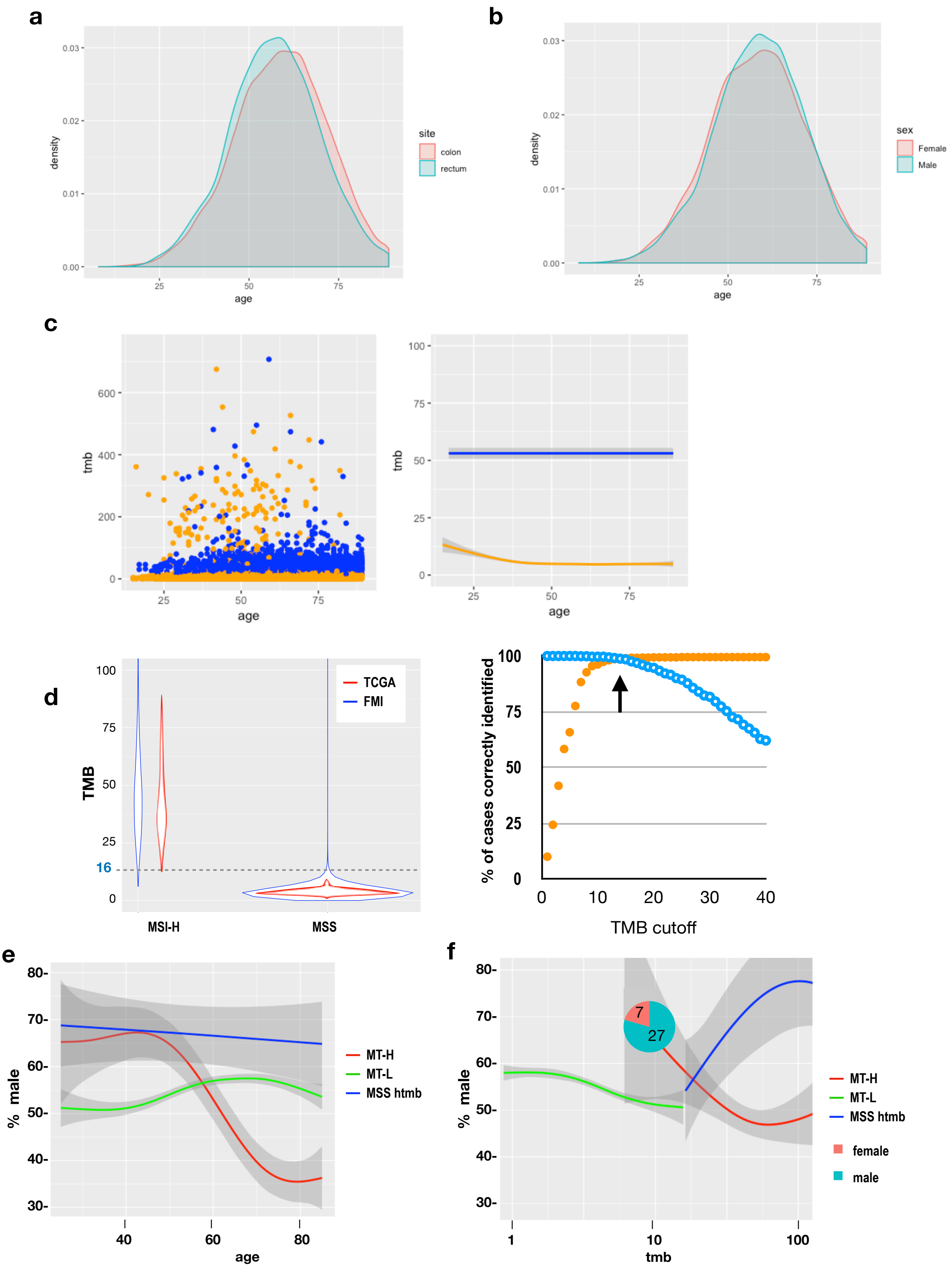

Serebriiskii et.al.

Supplementary Fig. 1. Additional characteristics of cohort, including analysis of relationship between TMB, age, sex, and tumor subsite (related to Fig.1; Source Data are provided as a Source Data file**)**. **a, b**. Age distribution for patients with sequenced tumors arising in the colon versus rectum (**a**), or in males versus females (**b**). **c**. Distribution of TMB in relation to age in MSI-H and MSS tumors. Left, individual tumors; right, trend lines; gray shading indicates 95% confidence limit. Orange, MSS; blue, MSI-H. **d**. Establishing TMB cutoff score for assignment of likely MS status. Left, distribution of TMB in MSI-H and MSS tumors, for FMI (blue) and TCGA CRC (red) cohorts. Right, percentage of MSS (orange) versus MSI-H (blue) tumors correctly identified by a TMB cutoff. **e, f**. Gender ratio, plotted as % of males, in MT-L vs MT-H vs MSS-htmb tumors, based on age (**e**) or TMB (**f**). Inset, gender ratio in the MT-H subset with the TMB<16. Gray shading indicates 95% confidence interval.

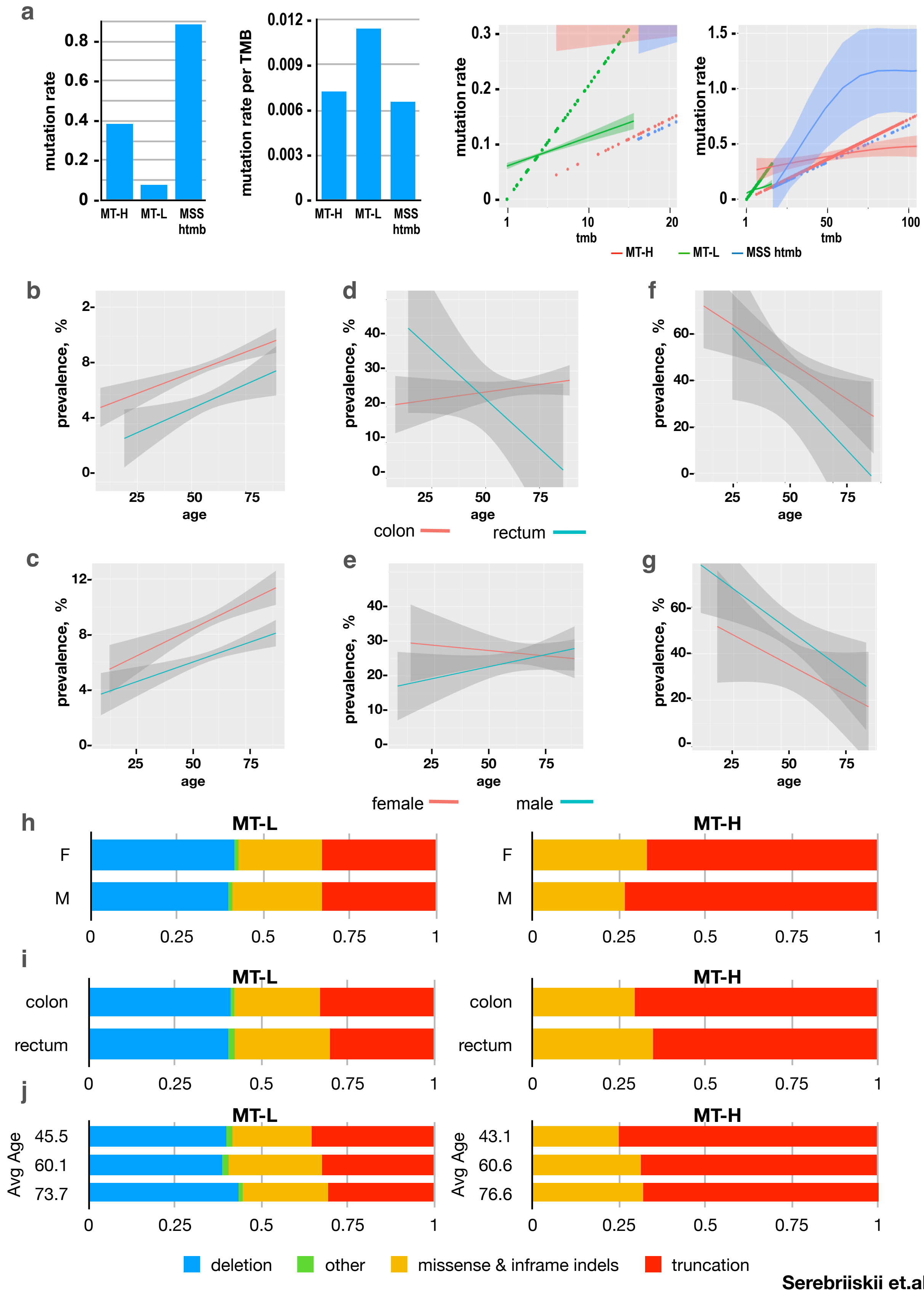

Serebriiskii et.al.

Supplementary Fig. 2. Patterns of *PTEN* mutation based on age, sex, and tumor subsite, in MT-L, MT-H, and MSS-htmb tumors (related to Fig.2; Source Data are provided as a Source Data file**)**. **a**. Frequency of alteration of *PTEN* in MT-L, MT-H, or MSS-htmb subsets (left), and normalized to TMB (second panel from left). Right two panels: actual (solid line) number of *PTEN* mutations observed at distinct levels of TMB, versus number predicted if mutations occur exactly proportional to TMB (dotted line); tumors with low TMB are shown in panel second from right, and with high TMB at far right. Shading indicates confidence level of 95%. **b-g**. Percent tumors with at least one *PTEN* alteration based on tumor subsite (**b, d, f**) or sex (**c, e, g**) as a factor of age in MT-L (**b, c**), MT-H (**d, e**), or MSS-htmb (**f, g**) CRC. Shaded areas represent 95% confidence intervals. Sample sizes, logistic regression coefficients and exact p-values are provided in Supplementary Tables 8 and 9. **h-j**. Percentage of *PTEN* alterations falling into the indicated classes based on sex (**h**), tumor subsite (**i**), or age (**j**) in MT-L or MT-H CRC. Blue, deep deletion; red, truncation (frameshift, nonsense); yellow, missense or small indel; green, other.

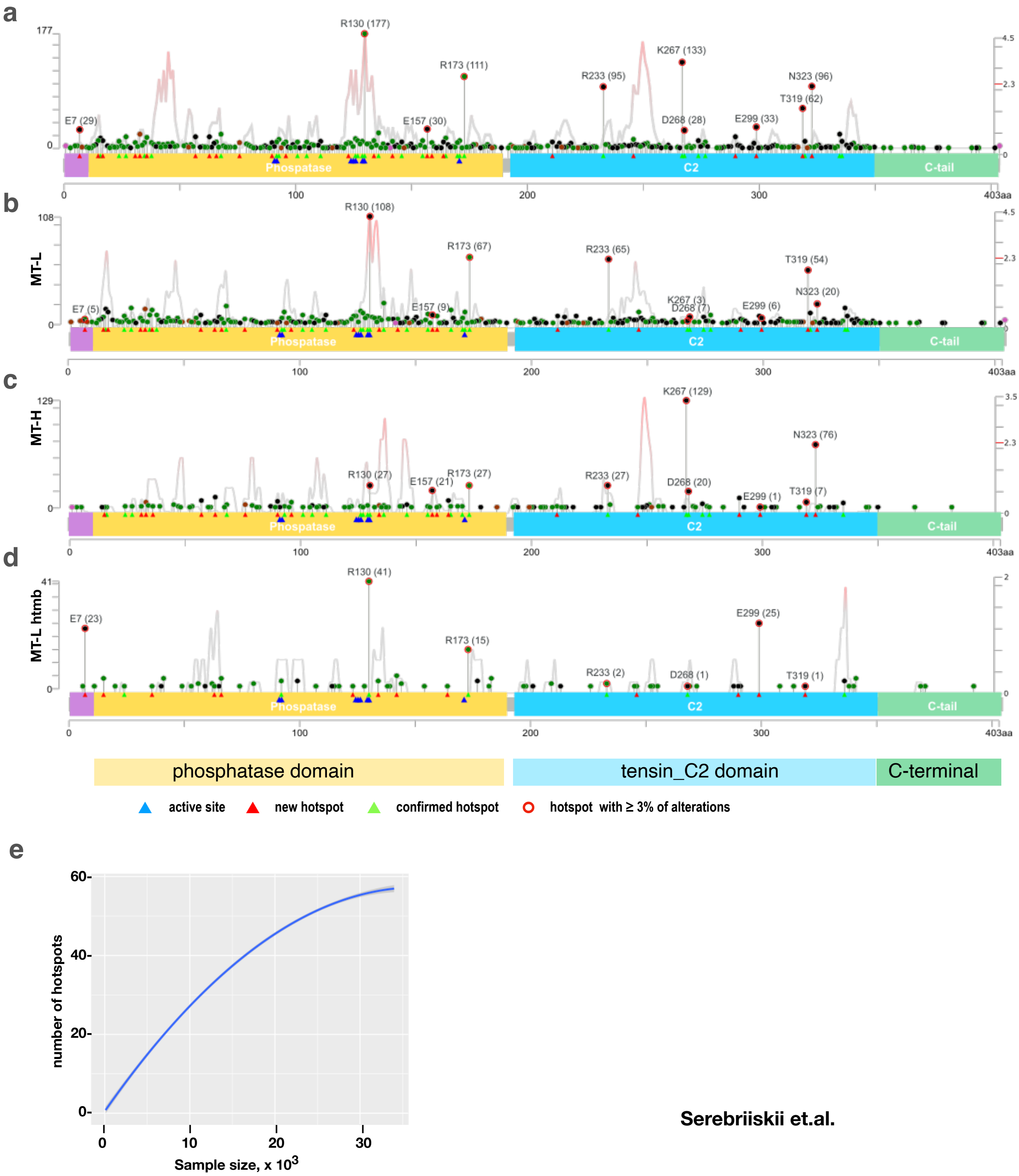

Serebriiskii et.al.

Supplementary Fig. 3. ***PTEN* mutations identified in CRC cohort (related to Fig.3)**. Location of all mutations of all classes, including truncating mutations, identified in the complete cohort (**a**), or the MT-L (**b**), MT-H (**c**), or MSS-htmb (**d**) subsets. The height of each lollipop indicates the count of the corresponding mutation in the dataset (left Y-axis). Red triangles underneath the lollipops: novel hotspots identified in this work; green triangles, previously hotspots present in the dataset; red circles on lollipops, hotspots representing >3% of total mutations observed in at least one subset. Density distribution (light gray line) represents the probability of concentration of non-hotspot mutations along the primary structure of *PTEN*, and is plotted as -log10(p) on the right Y-axis, with the values above the indicated 2.3 threshold corresponding to p-values <0.005. Mutation counts for each codon and p-values for sliding window analysis are provided as a Source Data file. **e.** Hotspots potentially identifiable in *PTEN* based on analysis of number of sequenced CRC tumors, as described in [1]; the current analysis has identified 54 hotspots. Shaded areas represent 95% confidence intervals. Hotspot mutations and mutation-enriched stretches along the primary protein sequence were identified using a binominal distribution model with a p-value cutoff 0.005, uncorrected for multiple comparisons. Source Data are provided as a Source Data file.

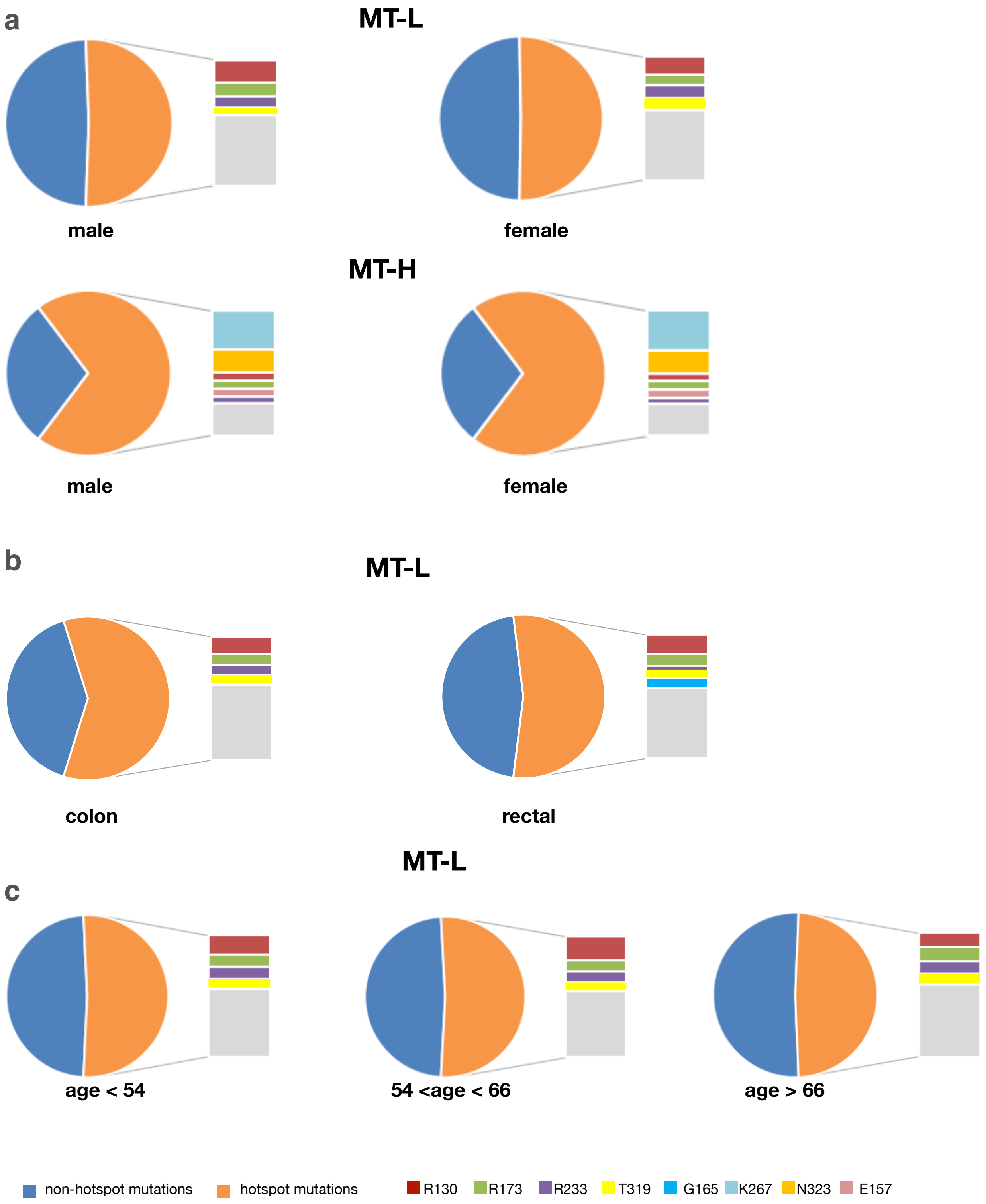

Serebriiskii et.al.

Supplementary Fig. 4. *PTEN* hotspot mutation analyzed based on sex, tumor subsite, or age (related to Fig.3). **a-c.** Pattern of codon location of hotspot mutations in MT-L or MT-H tumors as indicated, analyzed by sex (**a**), or age as subdivided into groups with equal sample size (**b**), or tumor subsite (**c**). No difference in patterns was identified, using chi-squared contingency table test. Insufficient cohort size limited this detailed analysis of hotspot mutations in the MSS-htmb and (in some cases) MT-H cohorts.

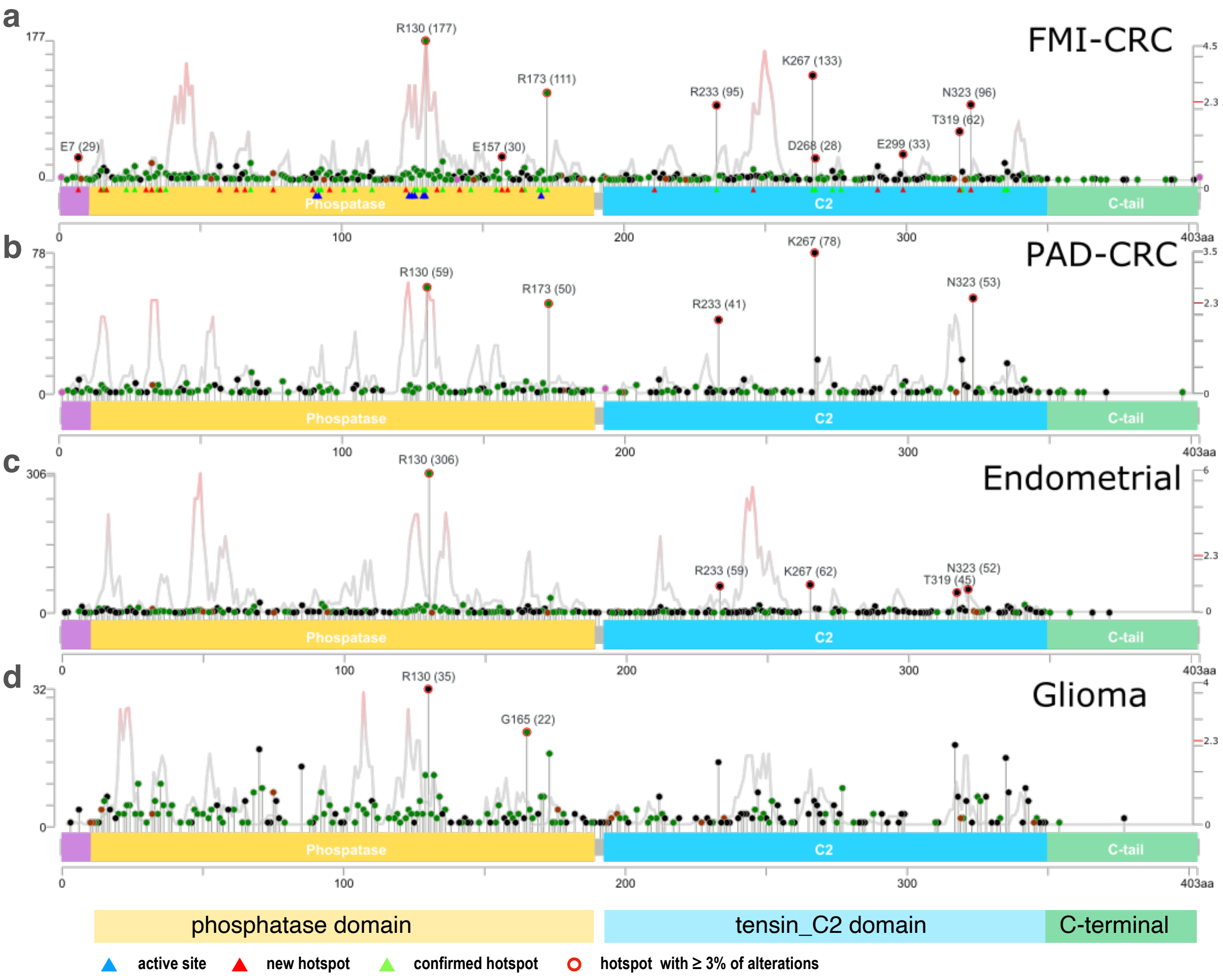

Serebriiskii et.al.

Supplementary Fig. 5. Comparison of *PTEN* mutations observed in FMI CRC cohort versus publicly available data for CRC and other tumor types (related to Fig.3). Location of mutations in *PTEN* protein identified in this study (**a**) contrasted to patterns identifiable from CRC in publicly available data (PAD-CRC) (**b**), or to two cancer types with high frequency of *PTEN* mutation, endometrial and glioma, based on data in AACR GENIE (**c, d**). The height of each lollipop indicates the count of the corresponding mutation in the dataset (left Y-axis). Red circles on lollipops, hotspots representing >3% of total mutations observed in at least one subset. Density distribution (light gray line) represents the probability of statistically significant concentration of non-hotspot mutations along the primary structure of *PTEN*, and is plotted as -log10(p) on the right Y-axis, with the values above the indicated 2.3 threshold corresponding to p-values below 0.005. Hotspot mutations and mutation-enriched stretches along the primary protein sequence were identified using a binominal distribution model with a p-value cutoff 0.005, uncorrected for multiple comparisons. Mutation counts for each codon and p-values for sliding window analysis are provided as a Source Data file.

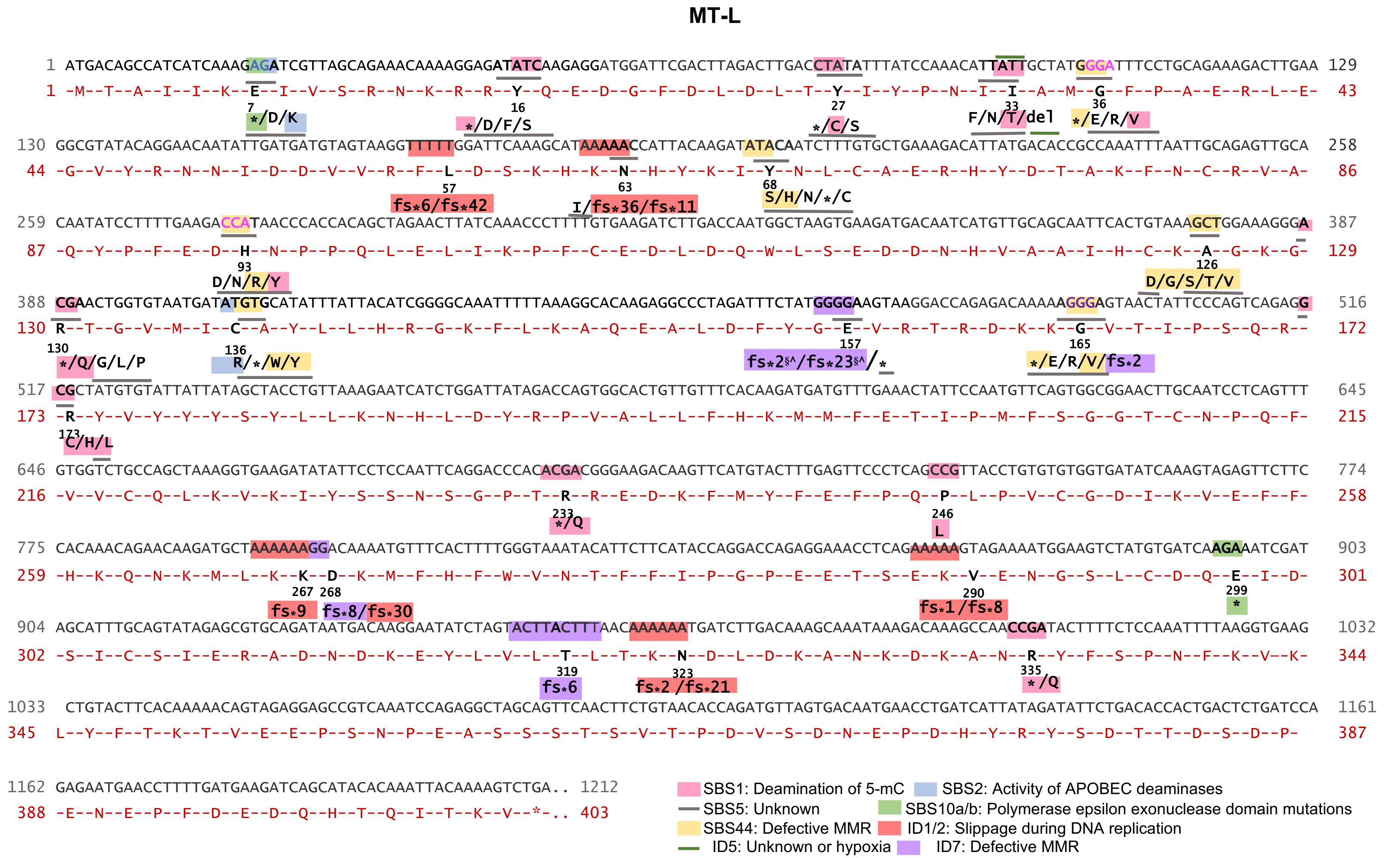

Serebriiskii *et. al.*

Supplementary Fig. 6a. Mutational signatures associated with *PTEN* hotspots (related to Fig.4). Nucleotide sequences annotated with mutation signatures MT-L tumor subset. Amino acids shown under lines represent protein changes associated with distinct mutational processes.

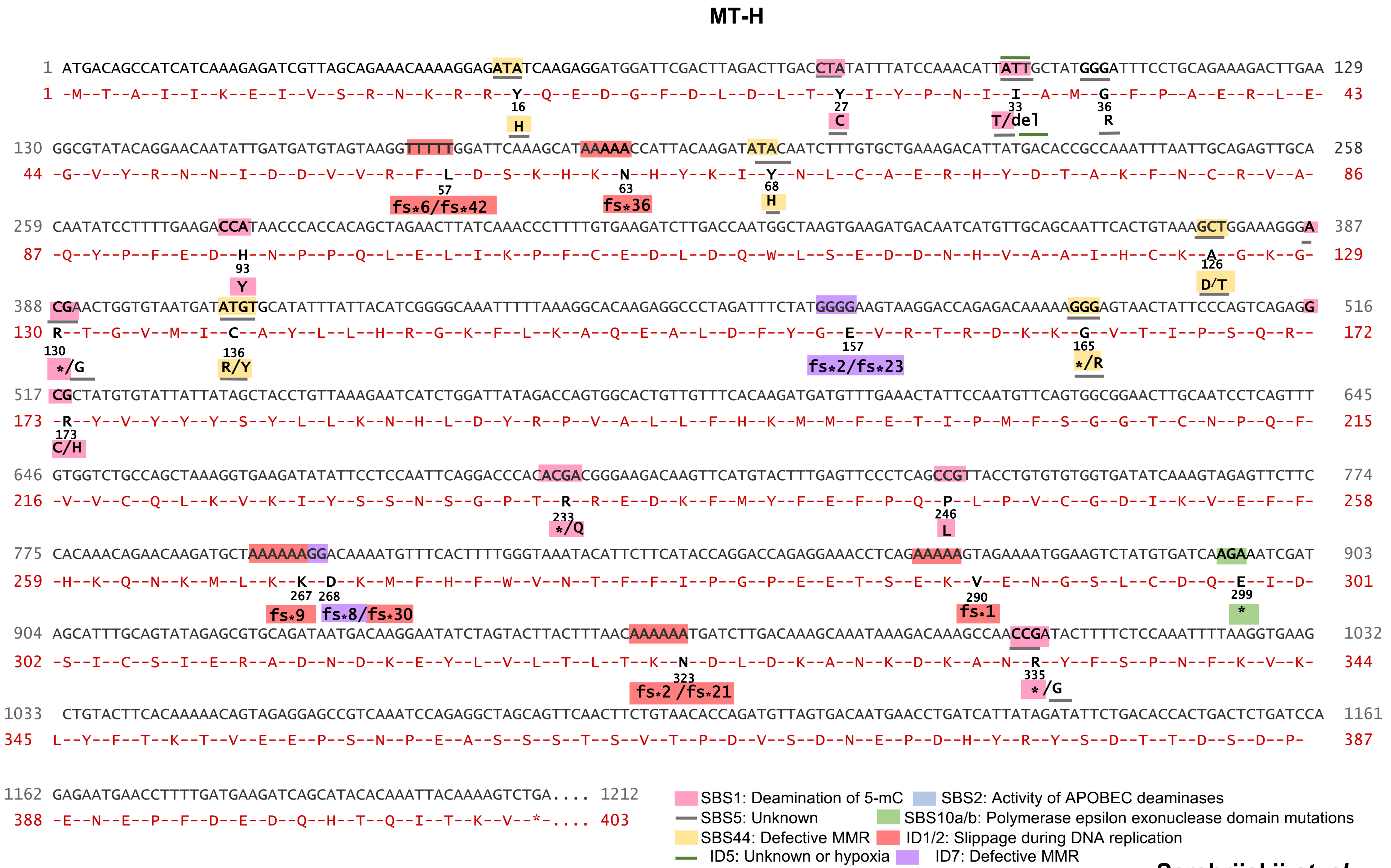

Serebriiskii *et al.*

Supplementary Fig. 6b. Mutational signatures associated with *PTEN* hotspots (related to Fig.4). Nucleotide sequences annotated with mutation signatures for MT-H tumor subset. Amino acids shown under lines represent protein changes associated with distinct mutational processes.

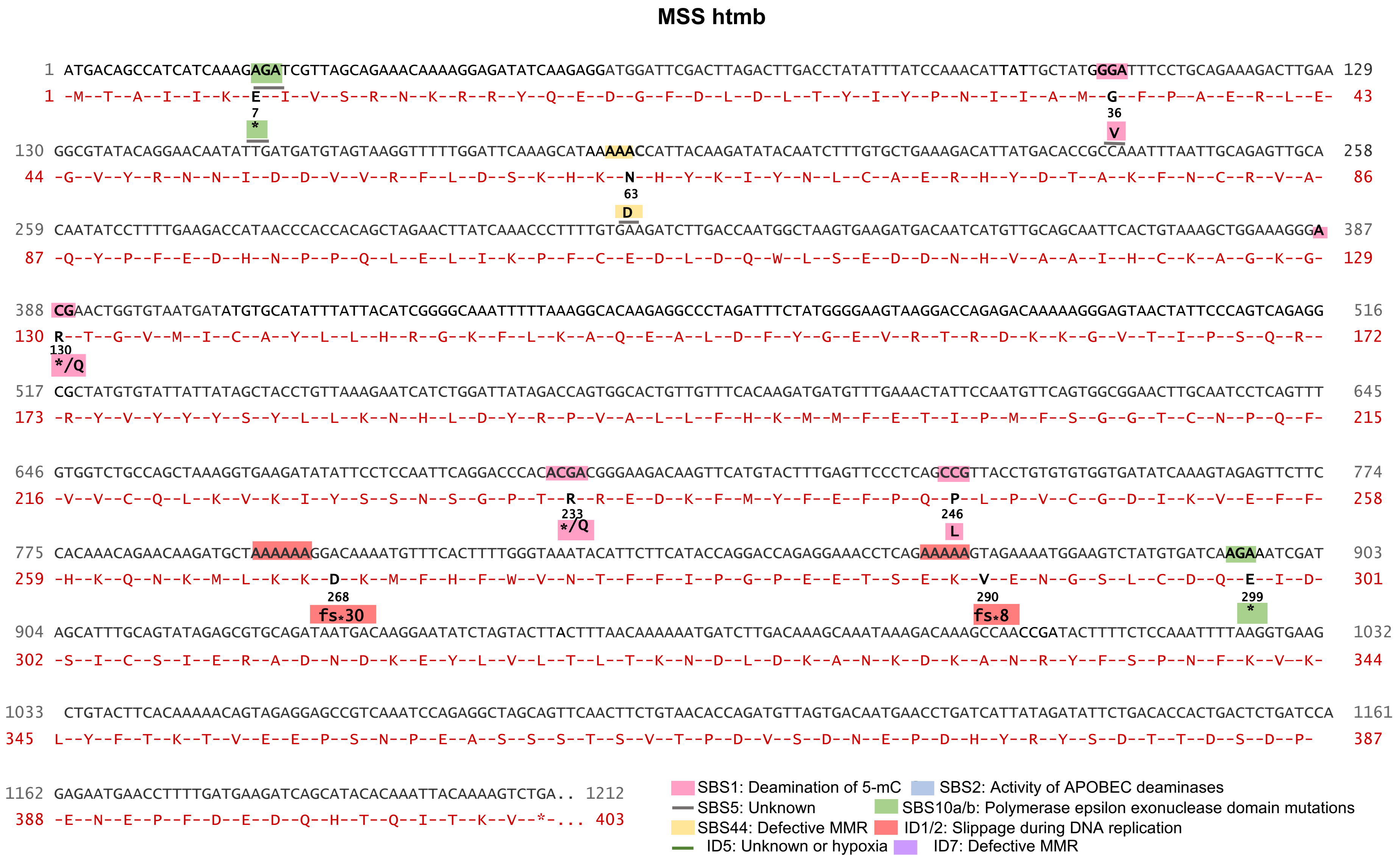

Serebriiskii *et. al.*

Supplementary Fig. 6c. Mutational signatures associated with *PTEN* hotspots (related to Fig.4). Nucleotide sequences annotated with mutation signatures for MSS htmb tumor subset. Amino acids shown under lines represent protein changes associated with distinct mutational processes.

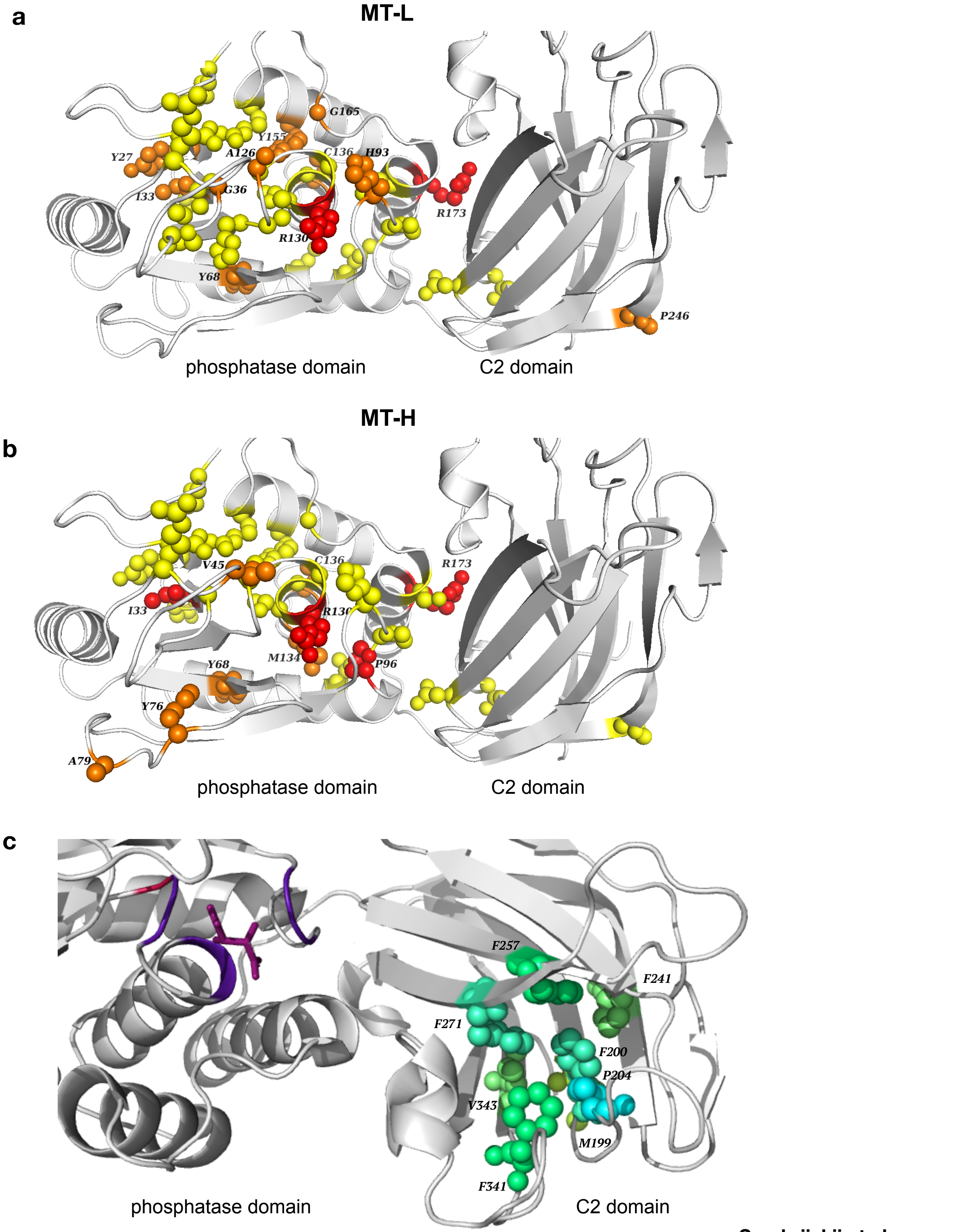

Serebriiskii et.al.

Supplementary Fig. 7. Location of selected hotspot missense and indel mutations in MT-H versus MT-L tumors, and of a 3D hotspot in the C2 domain on PTEN 3D structure (modeled from pdb:1D5R [https://www.rcsb.org/structure/1d5r]; related to Fig.5). a., b. MT-H **(a)** and MT-L **(b)** missense and indel mutations. Heatmap scale for MT-L**:** Yellow, counts >6 (R15, D24, N31, M35, P38, R47, P95, I101, C105, H123, C124, G127, G129, T131, K128, G132, R159, Q171, D252, T277); Orange, counts > 10 (Y27, I33, G36, Y68, H93, A126, C136, Y155, G165, P246); Red, R130 and R173. Heatmap scale for MT-H**:** Red, 7 or more (I33, P96, R130, R173); Orange, 3 or 4 (V45, Y68, Y76, A79, M134, C136); Yellow, 1 or 2 (R15, D24, Y27, N31, G36, R47, H93, P95, I101, A126, G127, G129, G132, Y155, R159, G165, S170, P246, D252, T277). **c.** 3D hotspot identified in C2 domain. Heatmap scale for number of mutations: 5**,** lime green **(**F257 and F341); 4, cyan, (P204); 3, limon (M199); 2, lime (V343 and F241); 1, green cyan (F200 and F271) (color names according to PyMol specifications).

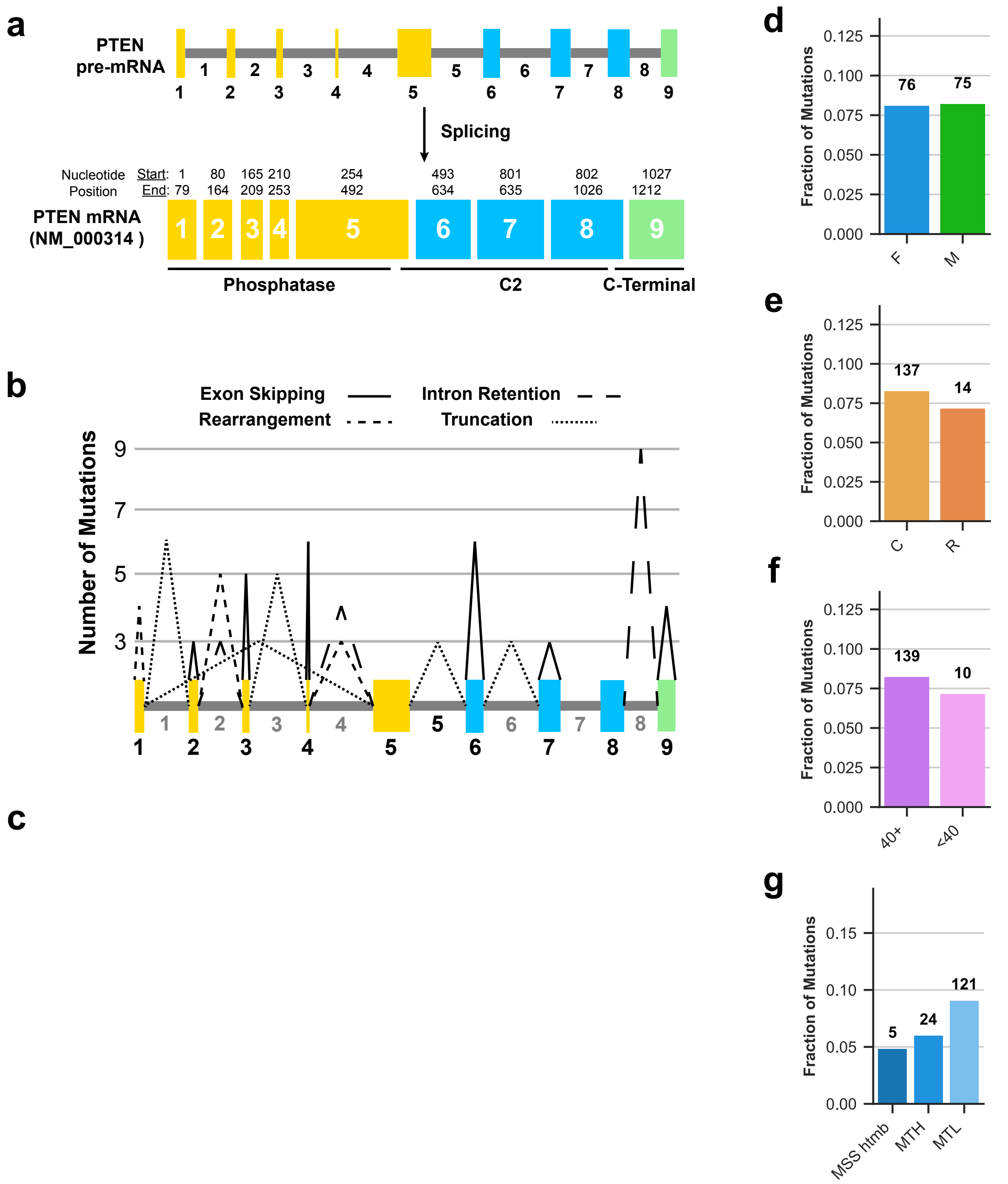

Serebriiskii *et.al.*

Supplementary Fig. 8. PTEN mutations affecting splicing. a. Nucleotide positions of introns and exons in PTEN with Human Genome Sequence Variant (HGVS) nomenclature for donor and acceptor splice site mutations (**a**). Number of mutations by intron (**b**) and +2, +1, -1, -2 positions (**c**). Fraction of PTEN splice mutations by sex (**d**), tumor site (**e**), age (**f**), and in the MT-L, MT-H, and MSS-htmb cohorts (**g**). No statistically significant relationship between mutations and patient characteristics (**d-g**) was found, as assessed by a two-sided Fisher exact test ($p > 0.05$).

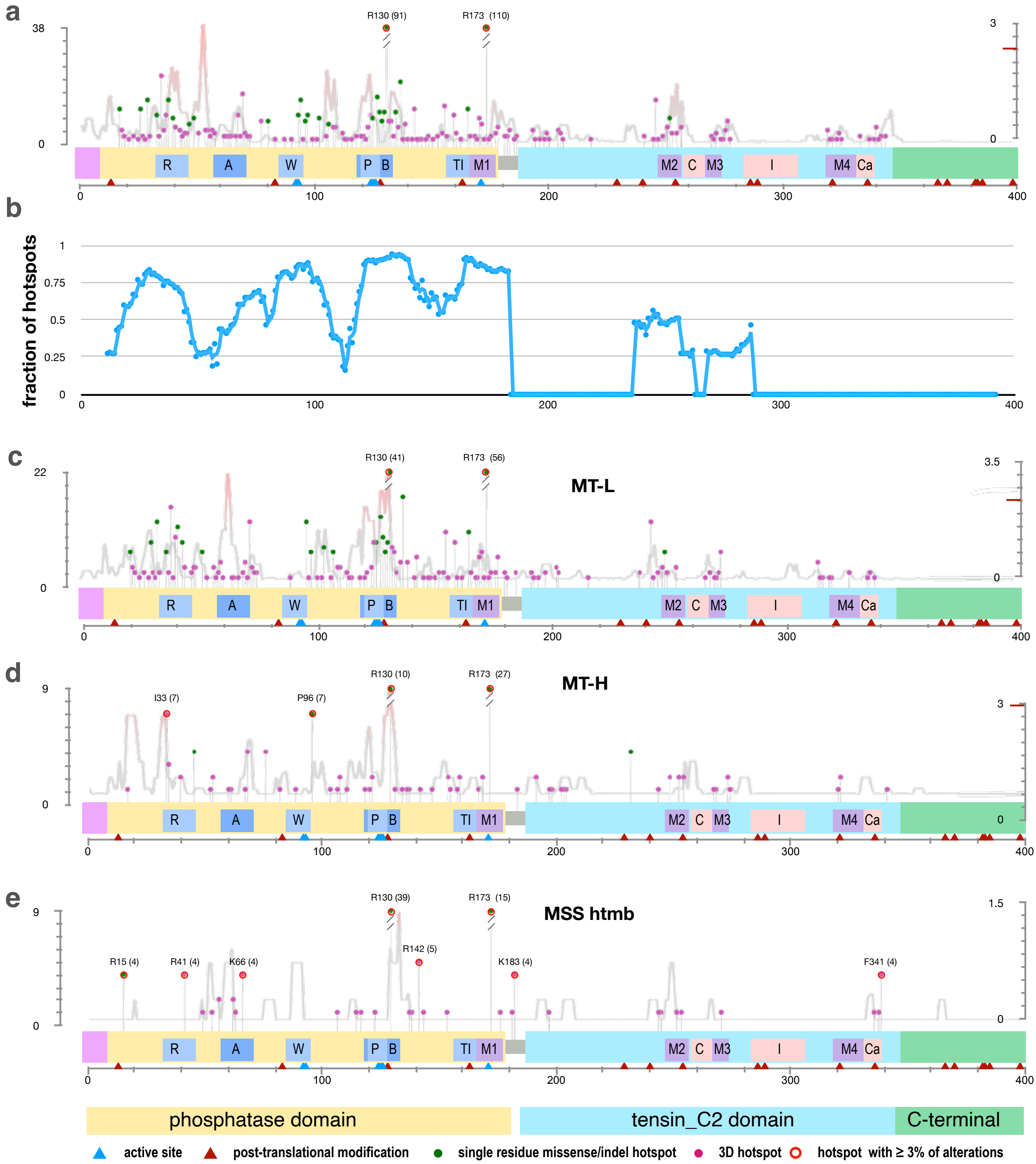

Supplementary Fig. 9. Missense and small indel mutation hotspots affecting protein sequence in *PTEN* (related to Fig.5). a. Location of missense/small indel hotspot mutations in *PTEN* protein identified in this study (green; >6 mutations observed), and 3D hotspots (magenta) in complete set of CRCs. Hotspots that are members of both groups (>6 mutations but located in a region of elevated 3D mutation) are colored magenta. **b.** Fraction of single-residue hotspot mutations based on sliding window analysis (window size: 20aa). **c-e.** Location of missense and small indel hotspots (green) and 3D hotspots (magenta) in the MT-L (**c**), MT-H (d), and MSS-htmb (**e**) subsets. Description of protein features for **a**, and **c-e: R**, Arginine loop (35-49); A, ATP-binding type-A motif (60–73);W, WPD loop (88–98); P, P loop (123–131); B, ATP-binding type-B motif (122–136); TI, TI loop (160–171); M1, Inter-domain Motif 1 (169–180); M2, Inter-domain Motif 2 (250–259); C, CBR3 loop (260–269); M3, Inter-domain Motif 3 (264–276); I, Internal loop in C2 domain (286–309); M4, Inter-domain Motif 4 (321–334); Cα, Cα2 loop (321–342). Blue triangles, active site (aa 92,93,124-126,129,130,171); brown triangles, most common post-translational modifications. Hotspot mutations and mutation-enriched stretches along the primary protein sequence were identified using a binominal distribution model; significance of the enrichment of mutations in 3D hotspots was calculated in permutation-based test. P-value cutoff 0.005 was used as a threshold for hotspot detection, uncorrected for multiple comparisons. Missense and inframe-indel mutation counts for each codon and p-values for sliding window analysis are provided as a Source Data file.

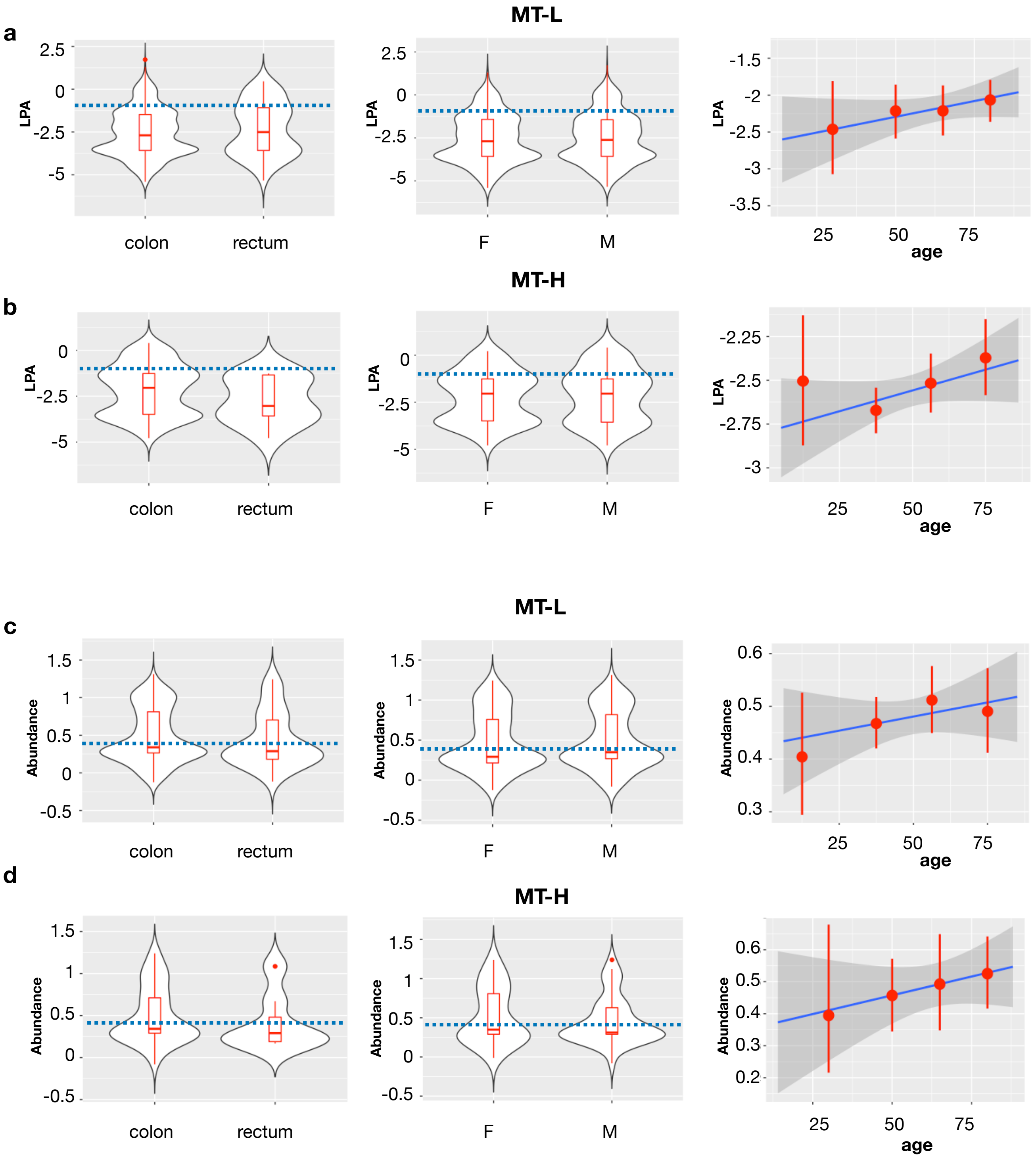

Serebriiskii et.al.

Supplementary Fig. 10. Lipid phosphatase activity (LPA) and protein abundance of *PTEN* mutations based on tumor subsite, sex, and age, in MT-L and MT-H CRC. **a-d**. LPA (**a, b**) and abundance (**c, d**) profiles for *PTEN* variants from MT-L **(a, c**) and MT-H (**b, d**) CRCs, based on colon versus rectum tumor subsite (left) or sex (center), or age (right). Box plots indicate median (middle line), 25th, 75th percentile (box) and 5th and 95th percentile (whiskers). Shaded areas represent 95% confidence intervals. Sample sizes for the violin plots are provided in Supplementary Tables 21, 22; no significant differences were found by sex, subsite (using Welch's unequal variances t-test and Kolmogorov-Smirnov tests) or age (using regression analysis).

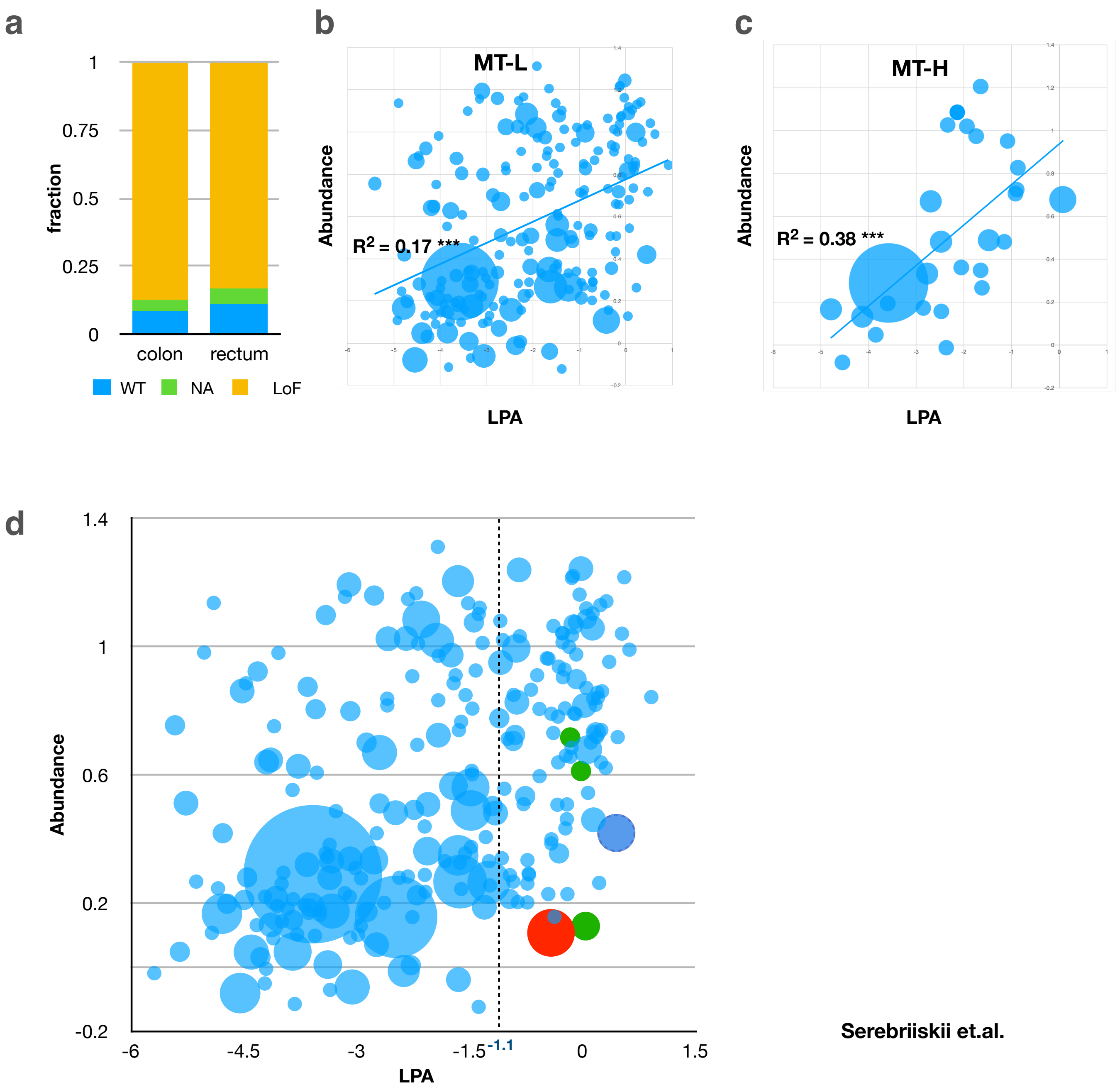

Serebriiskii et.al.

Supplementary Fig. 11. Frequency of mutations leading to distinct loss of function (LoF) profiles, based on abundance or lipid phosphatase activity (LPA) (related to Fig.6). **a.** *PTEN* LoF profile based on tumor subsite. WT, wild type; NA, not assigned due to lack of information; LoF, loss of function. Difference in LoF profile is not statistically significant, as determined by chi-squared contingency table test. **b, c.** LPA and abundance (VAMP-seq) profiles in the MT-L (**b**) and MT-H (**c**) subsets of CRC. *** indicates p-value < 2.2e-16 for MT-L subset, and 1. 8e-12 for MT-H subset, based on Pearson's product moment correlation coefficient (cor.test function in R, two-sided). Source Data are provided as a Source Data file. **d.** Defective activity reported for selected hotspot mutations that do not compromise LPA. *PTEN* Y336* (red) has reduced abundance but LPA comparable to wt *PTEN*; although this C-terminally truncated *PTEN* mutant does not function as a tumor suppressor, tumors bearing this mutation are sensitive to chemotherapeutic agents, and have altered heterochromatin structure [2]. Multiple mutations at K66 are observed (including K66N, K66T, K66M, shown in green), with different consequences on protein abundance and little effect on LPA. Notably, K27-linked ubiquitination of *PTEN* at K66 converts the protein from a phosphoinositide/Tyr phosphatase to a Ser/Thr phosphatase, suggesting these mutations may be selected based on loss of Ser/ Thr activity [3]. Some other mutations with limited effect on LPA, such as a recurring R142W mutation (purple), has been classified as “likely pathogenic” based on reduced ability to rescue the spheroid formation phenotype of normal mammary epithelial cells lacking *PTEN* [4]. For other common mutations, functional significance remains unknown.

a

MT-H Male (294) — # of copies altered

copy #	1	2	3	4	5
1	3%	0%	0%	0%	0%
2	48%	2%	0%	0%	0%
3	5%	7%	0%	0%	0%
4	0%	27%	0%	1%	0%
5	0%	3%	2%	0%	0%

MT-H Female (307) — # of copies altered

copy #	1	2	3	4	5
1	4%	0%	0%	0%	0%
2	56%	5%	0%	0%	0%
3	7%	7%	2%	0%	0%
4	0%	18%	0%	0%	0%
5	0%	1%	1%	0%	1%

MT-H Colon (485) — # of copies altered

copy #	1	2	3	4	5
1	1%	0%	0%	0%	0%
2	47%	2%	0%	0%	0%
3	10%	11%	1%	0%	0%
4	1%	16%	1%	0%	0%
5	0%	4%	3%	1%	1%

MT-H Rectum (46) — # of copies altered

copy #	1	2	3	4	5
1	0%	0%	0%	0%	0%
2	46%	4%	0%	0%	0%
3	11%	15%	2%	0%	0%
4	0%	17%	0%	0%	0%
5	2%	0%	2%	0%	0%

b

MT-L Male (8615) — # of copies altered

copy #	1	2	3	4	5
1	2%	0%	0%	0%	0%
2	23%	7%	0%	0%	0%
3	12%	17%	4%	0%	0%
4	3%	15%	4%	2%	0%
5	1%	3%	3%	2%	2%

MT-L Female (7802) — # of copies altered

copy #	1	2	3	4	5
1	2%	0%	0%	0%	0%
2	24%	8%	0%	0%	0%
3	12%	17%	4%	0%	0%
4	2%	14%	3%	2%	0%
5	1%	3%	3%	2%	2%

MT-L Colon (13740) — # of copies altered

copy #	1	2	3	4	5
1	2%	0%	0%	0%	0%
2	23%	8%	0%	0%	0%
3	12%	18%	4%	0%	0%
4	3%	14%	4%	2%	0%
5	1%	3%	3%	2%	2%

MT-L Rectum (2687) — # of copies altered

copy #	1	2	3	4	5
1	2%	0%	0%	0%	0%
2	24%	7%	0%	0%	0%
3	13%	15%	4%	0%	0%
4	2%	16%	3%	1%	0%
5	1%	4%	4%	2%	2%

c

MT-L htmb Male (97) — # of copies altered

copy #	1	2	3	4	5
1	0%	0%	0%	0%	0%
2	36%	3%	0%	0%	0%
3	11%	11%	0%	0%	0%
4	0%	19%	3%	0%	0%
5	0%	7%	7%	2%	0%

MT-L htmb Female (55) — # of copies altered

copy #	1	2	3	4	5
1	0%	0%	0%	0%	0%
2	38%	0%	0%	0%	0%
3	16%	13%	4%	0%	0%
4	7%	11%	7%	2%	0%
5	0%	0%	0%	2%	0%

MT-L htmb Colon (175) — # of copies altered

copy #	1	2	3	4	5
1	1%	0%	0%	0%	0%
2	56%	2%	0%	0%	0%
3	7%	2%	0%	0%	0%
4	1%	26%	0%	0%	0%
5	0%	3%	3%	0%	0%

MT-L htmb Rectum (27) — # of copies altered

copy #	1	2	3	4	5
1	0%	0%	0%	0%	0%
2	19%	4%	0%	0%	0%
3	7%	26%	0%	0%	0%
4	0%	30%	0%	0%	0%
5	0%	0%	15%	0%	0%

Serebriiskii *et.al.*

Supplementary Fig. 12. Copy number pattern of PTEN mutations by tumor sub-site and sex (related to Fig.7). a, b. For males versus females (**a**) or colon versus rectum subsites (**b**), data indicate co-occurrence of PTEN mutations with altered copy number of PTEN alleles. A value of 1 indicates loss of one allele; values of 3 or higher indicate increased gene copy number. Vertical axis, the estimated total PTEN copy number; on horizontal axis, the estimated copy number for the allele carrying PTEN mutations. Numbers in the cells indicate percent of all mutations with a combination of total/altered copy numbers. Source Data are provided as a Source Data file.

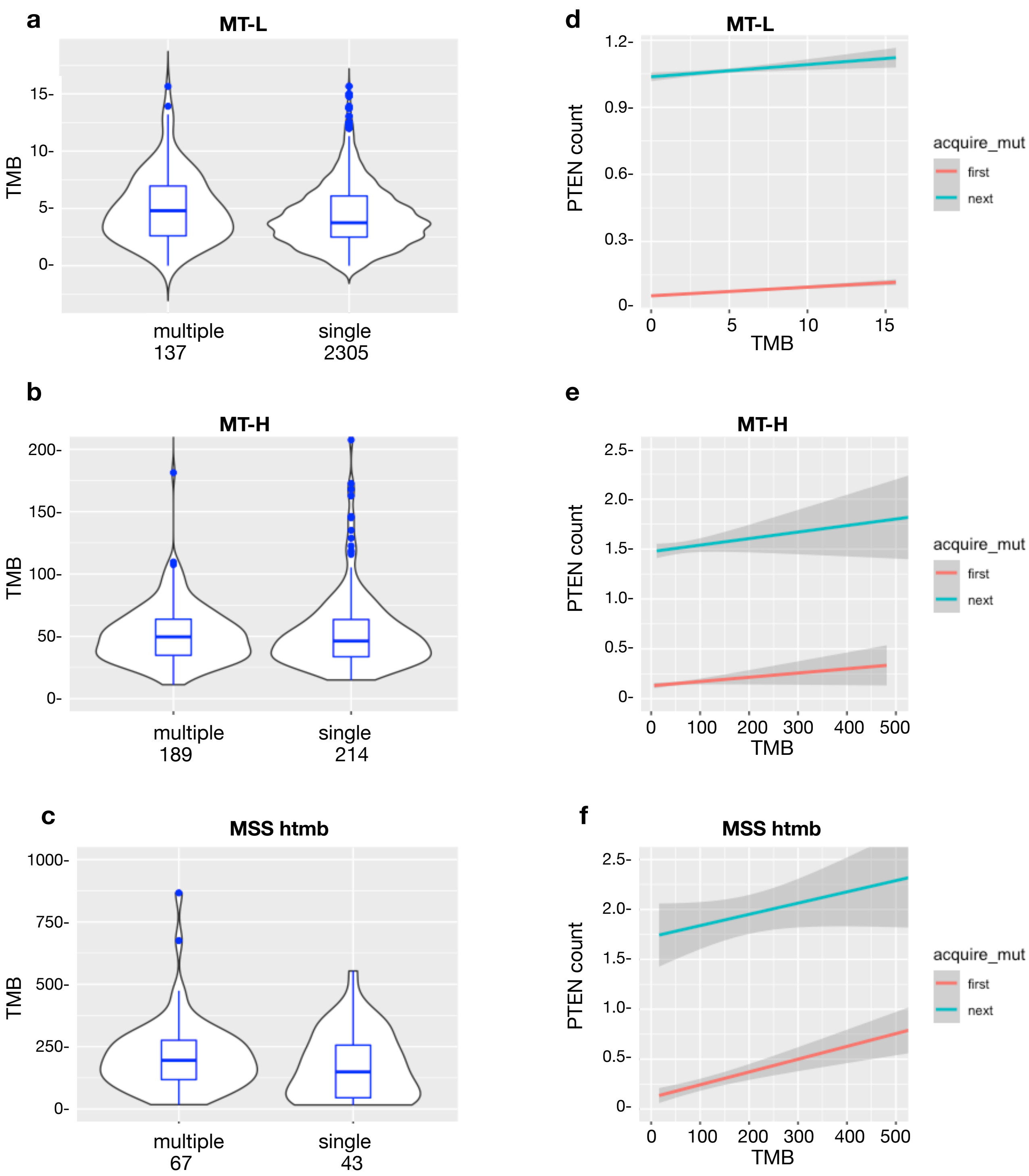

Serebriiskii et.al.

Supplementary Fig. 13. Relationship between multiplicity of *PTEN* mutations and TMB (related to Fig.7). **a-c**. Distribution of TMB based on the number of *PTEN* mutations in MT-L, MT-H, or MSS-htmb. No statistically significant difference was found using Welch's unequal variances t-test. Box plots indicate median (middle line), 25th, 75th percentile (box) and 5th and 95th percentile (whiskers). Number of samples for each plot is shown beneath the labels on X-axis. **d-f**. Frequency of *PTEN* alterations as a factor of TMB and multiplicity. Red, single *PTEN* alteration; blue-green, additional *PTEN* alterations. Shaded areas represent 95% confidence intervals. Source Data are provided as a Source Data file.

Supplementary references

1. Chang MT*, et al.* Accelerating Discovery of Functional Mutant Alleles in Cancer. *Cancer Discov* **8**, 174-183 (2018).

2. Chao JT*, et al.* A Premalignant Cell-Based Model for Functionalization and Classification of *PTEN* Variants. *Cancer Res* **80**, 2775-2789 (2020).

3. Hu Q*, et al.* LncRNAs-directed *PTEN* enzymatic switch governs epithelial-mesenchymal transition. *Cell Res* **29**, 286-304 (2019).

4. Gong L*, et al.* Nuclear *PTEN* tumor-suppressor functions through maintaining heterochromatin structure. *Cell Cycle* **14**, 2323-2332 (2015).

5. Post KL*, et al.* Multi-model functionalization of disease-associated *PTEN* missense mutations identifies multiple molecular mechanisms underlying protein dysfunction. *Nat Commun* **11**, 2073 (2020).
