# Supplementary material for: Comprehensive characterization of PTEN mutational profile in a series of 34,129 colorectal cancers
Source: Nat Commun. 2022 Mar 25;13:1618. doi: 10.1038/s41467-022-29227-2 (PMC8956741; doi:10.1038/s41467-022-29227-2)
Supplement: Supplementary file 3 — Description of Additional Supplementary Files [file 41467_2022_29227_MOESM3_ESM.pdf]

Description of Additional Supplementary Files

Supplementary Data 1

Description: Tables with summary level data for the studied dataset, including information on disease ontology, age, sex, tumour mutational burden, and MSI status.

Supplementary Data 2

Description: Table listing the identified 3D hotspots, with the number of mutations identified in the complete CRC cohort for each aminoacid in the cluster

Supplementary Data 3

Description: List of coding PTEN mutations classified by functional effect, including lipid phosphatase activity, abundance, and annotations for pathogenicity/damaging effect in relevant databases.

Supplementary Data 4

Description: **Cytoscape files for interaction of *PTEN* mutations**. A compressed archive file for Cytoscape file "PTEN_multiple_mutations_co-occ.cys", for the visualization of the co-occurrence between multiple *PTEN* mutations. Network legend: Edge width reflects degree of significance. Green edge indicates presence of co-occurring *POLE* mutations; most of these co-mutations involve the MSS-htmb cohort. Black edge indicates co-occurrence between the mutations compatible with signatures characteristic for MMR deficiency (dMMR; either IDT or SB44). Mult. Mut – share of samples with a given mutation which co-occur with a second *PTEN* mutation. Node color: darker color corresponds to a higher fraction of double mutation for a given mutation. Node border: increased width and shift towards purple color indicate a higher mutation count in the examined set.

Supplementary Data 5

Description: **PyMol files visualizing hotspot mutations on the PTEN protein structure**. A compressed archive file with PyMol files for the visualization of single-residue hotspots on the PTEN structure PDB: 1D5R [https://www.rcsb.org/structure/1d5r]. File "0509-MTL.pse" visualizes hotspots identified in MT-L subset of the data (Heatmap scale: Yellow, mutation counts >6; Orange, counts >10; Red, R130 and R173). File "0509-MTH.pse" visualizes hotspots identified in the MT-H subset of the data (Heatmap scale: Yellow, 1 or 2 mutations; Orange, counts 3 to 4, Red: 7 or more). File "0509 complete set.pse" visualizes hotspots identified in the complete set of PTEN mutations (Heatmap scale: Yellow, counts >6; Orange, counts >10; Red, counts >90)
