# Supplementary material for: Comprehensive characterization of PTEN mutational profile in a series of 34,129 colorectal cancers
Source: Nat Commun. 2022 Mar 25;13:1618. doi: 10.1038/s41467-022-29227-2 (PMC8956741; doi:10.1038/s41467-022-29227-2)
Supplement: Supplementary file 9 — Reporting Summary [file 41467_2022_29227_MOESM9_ESM.pdf]

nature portfolio

Corresponding author(s): Erica Golemis

Last updated by author(s): Feb 21, 2022

Reporting Summary

Nature Portfolio wishes to improve the reproducibility of the work that we publish. This form provides structure for consistency and transparency in reporting. For further information on Nature Portfolio policies, see our Editorial Policies and the Editorial Policy Checklist.

Statistics

For all statistical analyses, confirm that the following items are present in the figure legend, table legend, main text, or Methods section.

n/a	Confirmed	
☐	☒	The exact sample size (*n*) for each experimental group/condition, given as a discrete number and unit of measurement
☐	☒	A statement on whether measurements were taken from distinct samples or whether the same sample was measured repeatedly
☐	☒	The statistical test(s) used AND whether they are one- or two-sided<br>*Only common tests should be described solely by name; describe more complex techniques in the Methods section.*
☐	☒	A description of all covariates tested
☐	☒	A description of any assumptions or corrections, such as tests of normality and adjustment for multiple comparisons
☐	☒	A full description of the statistical parameters including central tendency (e.g. means) or other basic estimates (e.g. regression coefficient) AND variation (e.g. standard deviation) or associated estimates of uncertainty (e.g. confidence intervals)
☐	☒	For null hypothesis testing, the test statistic (e.g. *F*, *t*, *r*) with confidence intervals, effect sizes, degrees of freedom and *P* value noted<br>*Give P values as exact values whenever suitable.*
☒	☐	For Bayesian analysis, information on the choice of priors and Markov chain Monte Carlo settings
☒	☐	For hierarchical and complex designs, identification of the appropriate level for tests and full reporting of outcomes
☐	☒	Estimates of effect sizes (e.g. Cohen's *d*, Pearson's *r*), indicating how they were calculated

Our web collection on statistics for biologists contains articles on many of the points above.

Software and code

Policy information about availability of computer code

Data collection	Data collection and annotation were described in detail in PMID:24142049 (Frampton et al 2013) in a proprietary data collection pipeline developed by FMI. Sequence data were processed using a customized analysis pipeline designed to accurately detect multiple classes of genomic alterations. All testing was done in a CLIA-certified, CAP-accredited laboratory.
Data analysis	Standard statistical tests were implemented using R version 4.0. No custom software was used. The code used for identification of single-residue hotspots, mutation-enriched regions, and 3D hotspots is deposited at Serebriiskii IG, Pavlov V, Andrianov G. Comprehensive characterization of PTEN mutational profile in a series of 34,129 colorectal cancers. Github repository; https://doi.org/10.5281/zenodo.6149413) (2022).

For manuscripts utilizing custom algorithms or software that are central to the research but not yet described in published literature, software must be made available to editors and reviewers. We strongly encourage code deposition in a community repository (e.g. GitHub). See the Nature Portfolio guidelines for submitting code & software for further information.

Data

Policy information about availability of data

All manuscripts must include a data availability statement. This statement should provide the following information, where applicable:

- Accession codes, unique identifiers, or web links for publicly available datasets
- A description of any restrictions on data availability
- For clinical datasets or third party data, please ensure that the statement adheres to our policy

Consented data that can be released for publication are included in the article and its supplementary files, and include permanently de-identified data on PTEN mutation status, the presence of mutations in other genes noted in the study, and sex, age, and tumor subsite for individuals profiled by FMI. Patients were not

consented for the publication of underlying sequence data, nor can published data describe raw sequence data or link sequence data to patient clinical phenotypes. We sent a proposal describing the scope of our work through the Foundation Medicine website and we then filed out a study review form, which was checked by lawyers at each end. After the approval of a data transfer agreement, Foundation Medicine assigned us with specialists in the dataset that were interested in colorectal cancer. Academic researchers can gain access to underlying Foundation Medicine data in this study by contacting Foundation Medicine using the coordinates on their website (https://www.foundationmedicine.com/contact), and filling out a data request form. Researchers and their institutions will be required to sign a data transfer agreement. The public web resources used in this paper are listed here: The cBioPortal for Cancer Genomics, https://www.cbioportal.org ; AACR Project GENIE, https://genie.cbioportal.org; the Catalogue Of Somatic Mutations In Cancer, https://cancer.sanger.ac.uk/cosmic; the Surveillance, Epidemiology, and End Results (SEER) Program, https://seer.cancer.gov. PyMol files for the visualization of hotspots on the PTEN structure (1D5R [https://www.rcsb.org/structure/1d5r]), and the Cytoscape file for the visualization of the co-occurrence between multiple PTEN mutations are provided with this paper as Supplementary Information Files (Supplementary Data 4.zip and Supplementary Data 5.zip). The remaining data are available within the Article, Supplementary Information or Source Data file.

Field-specific reporting

Please select the one below that is the best fit for your research. If you are not sure, read the appropriate sections before making your selection.

- [x] Life sciences
- [ ] Behavioural & social sciences
- [ ] Ecological, evolutionary & environmental sciences

For a reference copy of the document with all sections, see nature.com/documents/nr-reporting-summary-flat.pdf

Life sciences study design

All studies must disclose on these points even when the disclosure is negative.

Sample size	The dataset corresponded to the full set of colorectal cancers sequenced by FMI between 2015-2019 (~34,000), which defined the sample size
Data exclusions	Data missing some key clinical parameters were excluded from analyses relevant to that specific parameter.
Replication	There was no feasibility for performing a replication study. This was not possible because the dataset was based on the entire set of tumors sequenced over multiple years by a leading genomics company - there is no way to accrue a similar cohort without waiting another 4 years.
Randomization	This was not relevant to the analysis. We analyzed the entire, single, large sequencing data set as a group, so there was nothing to randomize.
Blinding	Not performed, and not relevant to the analysis. The types of comparison performed were enumeration of mutation frequencies within a single data set, so there was nothing to blind.

Reporting for specific materials, systems and methods

We require information from authors about some types of materials, experimental systems and methods used in many studies. Here, indicate whether each material, system or method listed is relevant to your study. If you are not sure if a list item applies to your research, read the appropriate section before selecting a response.

Materials & experimental systems

n/a	Involved in the study	
x		Antibodies
x		Eukaryotic cell lines
x		Palaeontology and archaeology
x		Animals and other organisms
x		Human research participants
x		Clinical data
x		Dual use research of concern

Methods

n/a	Involved in the study	
x		ChIP-seq
x		Flow cytometry
x		MRI-based neuroimaging
